# The Power of Partnership: Adapting Early Language Intervention for Children With Down Syndrome Through Family‐Researcher Collaboration

**DOI:** 10.1111/1460-6984.70139

**Published:** 2025-10-11

**Authors:** Kirstie Hartwell, Emma Pagnamenta, Vesna Stojanovik, Rebecca Baxter, Kelly Burgoyne

**Affiliations:** ^1^ Manchester Institute of Education University of Manchester Manchester UK; ^2^ School of Psychology and Clinical Language Sciences University of Reading Reading UK

**Keywords:** community‐based participatory research, design‐based research, Down syndrome, families, intervention, language

## Abstract

**Background:**

Parents are uniquely placed to support their child's development. Interventions which are designed to be delivered by parents therefore hold considerable promise, particularly for children with neurodevelopmental conditions that are associated with particular developmental strengths and challenges.

**Aims:**

This study worked in partnership with families from the Down syndrome community to adapt an evidence‐based early language intervention for children with Down syndrome.

**Methods and Procedures:**

Six families with a 3‐ to 5‐year‐old child with Down syndrome participated in this mixed‐methods exploratory study. Guided by aspects of Community‐Based Participatory Research and Design‐Based Research, iterative cycles of design, implementation, analysis, and re‐design were implemented to produce an adapted intervention programme. Data were collected using record forms, surveys, observations, and focus groups.

**Outcomes and Results:**

Findings showed many aspects of the original programme were acceptable and feasible for families, but important adaptations were identified, including enhancing repetition and consolidation, reducing time pressures, tailoring to individual needs, smaller steps for learning, supporting engagement, and increasing visual support. Adapting the programme in these ways enhanced adherence, enjoyment and the child's active engagement.

**Conclusions and Implications:**

This study is the first to report the process of adapting an existing language intervention for people with disabilities and highlights the value of working with families to identify the best ways to support their needs. Our approach shows promise for supporting language development in this population and serves as a foundation for future research that aims to develop novel interventions.

**WHAT THIS PAPER ADDS:**

*What is already known on the subject*

- Speech and language difficulties are well‐documented in individuals with Down syndrome, and may benefit from intervention to enable individuals to reach their full potential. Parent‐delivered models of intervention hold promise for supporting oral language skills, but very few evidence‐based interventions exist for this population.

*What this paper adds to the existing knowledge*

- Parents of young children with Down syndrome are able to deliver early language intervention in ways which encourage their child's active involvement and enjoyment. Parents identified a range of ways in which early language intervention could be adapted to support implementation and effectiveness.

*What are the potential or actual clinical implications for this work?*

- Parents are important partners in early language intervention for children with Down syndrome, with much to contribute to the design and delivery of intervention. Findings from this study will guide researchers and clinicians in the process of adapting interventions for this population, with the aim of improving outcomes for children and their families.

## 1 Introduction

The right to communicate is enshrined in Article 21 of the United Nations Convention on the Rights of Persons with a Disability (United Nations 2006), which sets out the responsibility of the state to ensure that people with disabilities have access to support for effective communication. Accessing support can, however, be challenging (Frizelle et al. 2022), particularly where overwhelming demand for speech and language therapy (SLT) services is coupled with a shortage of qualified therapists (Royal College of Speech and Language Therapists 2023) and a lack of evidence‐based interventions for language difficulties (e.g., Smith et al. 2020). This context calls for alternative models of intervention and support, and parent‐delivered interventions constitute a promising approach. Such interventions should account for aetiology‐specific context (Fidler et al. 2021) and ideally be designed in partnership with families to be feasible and acceptable to them (Walsh et al. 2024). This paper describes the process of working with families with children with Down syndrome to adapt an evidence‐based parent‐delivered early language intervention. Guided by aspects of Community‐Based Participatory Research (CBPR; Israel et al. 2010) and Design‐Based Research (DBR; Tinoca et al. 2022), we present data collected through two cycles of piloting and adaptation. To our knowledge, this is the first paper to report the process of adapting an existing language intervention for people with disabilities. This is an important learning which has implications for the development of other novel interventions.

### 1.1 Down Syndrome

Down syndrome is a genetic disorder that causes a range of developmental changes and physical features and is associated with heightened risk of some health conditions, including vision and hearing problems (Antonarakis et al. 2020). Whilst the severity of cognitive impairment is highly variable (IQ range 30–70; Chapman and Hesketh 2000), there is a pattern of strengths and weaknesses associated with the Down syndrome phenotype which can inform effective intervention (Fidler and Nadel 2007).

Whilst strengths in social communication and non‐verbal skills are common, speech and language impairments are characteristic of individuals with Down syndrome (Abbeduto et al. 2016). Receptive language is typically stronger than expressive language, which is impacted by delays in vocabulary development (Chapman and Hesketh 2000). Though many families support early vocabulary skills through manual signing (Frizelle and Lyons 2022), expressive delays often continue and lead others to underestimate an individual's abilities. Particular challenges are observed for grammar, verbal short‐term memory (Naess et al. 2015), speech clarity (Burgoyne et al. 2021), and narrative skills (Chapman and Hesketh 2000).

Within this broad profile, there are considerable individual differences attributable to a complex interplay of influences operating at multiple levels. At the environmental level, variability may result from differences in family patterns of interaction, including parent‐child communication, child experiences (e.g., inclusive education) and child health (Guralnick 2017). These factors are further influenced by resources, including parent characteristics (e.g., parent mental health and wellbeing), time, finances, and access to therapy and social support (Guralnick 2017). Environmental factors may play a larger role in shaping development for children with Down syndrome than for those developing typically (Abbeduto et al. 2016), making early intervention particularly important to maximise social competence and cognitive development (D'Souza et al. 2017; Guralnick 2017). Evidence suggests that intervention for speech, language and communication can be effective for children with Down syndrome but points to significant limitations with existing work and the need for further high‐quality intervention studies (Seager et al. 2022; Smith et al. 2020).

### 1.2 The Role of Parents in Early Language Development and Intervention

The quantity and quality of adult‐child communicative interactions play a significant role in shaping children's language development (Abbeduto et al. 2016; Golinkoff et al. 2019). Whilst child language varies as a function of parental input (Hoff et al. 2014), parental input itself is affected by the child's cognitive and language and communicative abilities, as well as particular features of the behavioural phenotype (Abbeduto et al. 2016; Zampini et al. 2012). For example, limited expressive language may result in fewer child‐initiated communicative interactions and may lead parents to unconsciously underestimate children's abilities (D'Souza et al. 2017). Such unconscious assumptions may in turn limit the learning opportunities children are exposed to (D'Souza et al. 2017); for example, parents may simplify and restrict their language input, thereby limiting exposure (Zampini et al. 2012). Changing the learning environment by providing parents with intervention may challenge assumptions and enhance children's learning opportunities, leading to new insights for parents and learning for children. As such, it has been suggested that combining naturalistic intervention strategies with direct structured intervention may optimise early language learning environments and lead to gains in language learning (McWilliam 2010; Warren et al. 2020). The evidence, however, of the effectiveness of parent‐mediated interventions for improving the language and communication of children with Down syndrome is inconclusive (O'Toole et al. 2018), and there is a need for well‐designed studies. An example of a parent‐delivered early language intervention which combines naturalistic strategies with direct intervention is Parents and Children Together (PACT) (Burgoyne et al. 2018).

### 1.3 The Parents and Children Together (PACT) Programme

Parents and Children Together (PACT) is a parent‐delivered early language intervention programme originally developed for typically developing preschool children at risk of early language delays. The programme provides a consistent structure of activities based around shared book reading. Shared book reading is a naturally occurring sociocultural activity which promotes children's early language development, particularly when parents use strategies that enhance the child's active participation and provide opportunities to model and scaffold language (Mol et al. 2008). The programme supplements shared book reading with direct work on vocabulary and narrative skills. A randomised controlled trial (RCT) conducted with 208 pre‐school children (aged 3 years) and their parents living in geographical areas of England with high levels of social deprivation demonstrated that children receiving PACT made significantly greater gains in language and early literacy than an active treatment control group (Burgoyne et al. 2018; though see Burgoyne et al. 2024).

PACT incorporates several features which support learning and development in people with Down syndrome, making it a potentially promising intervention for this group. The programme is social and interactive which make it motivating for children with DS (Fidler et al. 2006) and align with their relative strengths in social interaction (e.g., Abbeduto et al. 2016); it is highly visual, playing to the relative strengths in visual‐spatial memory of children with DS (Jarrold et al. 1999; Jarrold and Baddeley 1997); and it provides opportunities for repetition and consolidation (Chapman et al. 2000) which are essential in supporting learning as higher intensity leads to better outcomes (Buckley et al. 2024). Adaptations for individual strengths and weaknesses are encouraged, tailoring to the wide variability seen in Down syndrome. Finally, the structured routine, consisting of several short activities, supports attention and behaviour. Nonetheless, adaptations are likely necessary to support implementation and effectiveness for this population. Working in partnership with families provides a powerful approach to identifying and piloting those adaptations (Walsh et al. 2024).

### 1.4 Aims and Objectives of the Study

This exploratory study used aspects of CBPR and DBR to adapt the PACT programme for children with Down syndrome. The study worked closely with families from the Down syndrome community to achieve the following objectives:

1. Determine fidelity and parent experience of the original PACT programme.
2. Identify adaptations to the original PACT programme.
3. Determine fidelity and parent experience of an adapted (PACT‐DS) programme.

## 2 Method

### 2.1 Participants

The project was advertised via Down syndrome support groups and social media to recruit families with a 3–6 year old child with Down syndrome, living within 40 miles of the University of Manchester or the University of Reading. Children needed a minimum of 10 expressive words/signs, and parents needed to read and speak English to take part. The sample consisted of 6 children (5 boys; 1 girl), aged 3–5 years (mean age = 52.5 months, SD = 9.35). Parents (all mothers) reported that children's main form of communication was signing (*n *= 4), spoken language (*n *= 1) or sign and spoken language combined (*n *= 1). Ethical approval was granted by the University of Manchester Research Ethics Committee. Informed consent was obtained for all participants.

### 2.2 Materials and Procedure

#### 2.2.1 The PACT Programme

Parents and Children Together (PACT) is a parent‐delivered early language intervention programme. An overview of the teaching programme and an example of a teaching session are provided in Burgoyne et al. (2018) online Appendix C. Each intervention session follows a consistent structure and routine (see Table 1). Parents deliver the PACT programme with their child every day (5 days a week) for 20 min over 30 weeks (150 sessions in total). The programme is organised in 6 × 5‐week ‘Blocks’: In each Block, Weeks 1–4 introduce new learning and Week 5 focuses on revision and consolidation. Parents are given training and materials (books and resources) to carry out the sessions.

**TABLE 1:** Overview of PACT language session.

Component	Description	Duration
**Introduction**	Settle your child	2 min
**Reading Together**	Read the book together and talk about the story	5 min
**Vocabulary**	Play games with new words	5 min
**Stories**	Do activities together that focus on the key events in the story	5 min
**Reward**	Talk about what you did together and give your child a sticker	3 min

#### 2.2.2 Approach to Adaptation

**FIGURE 1:**
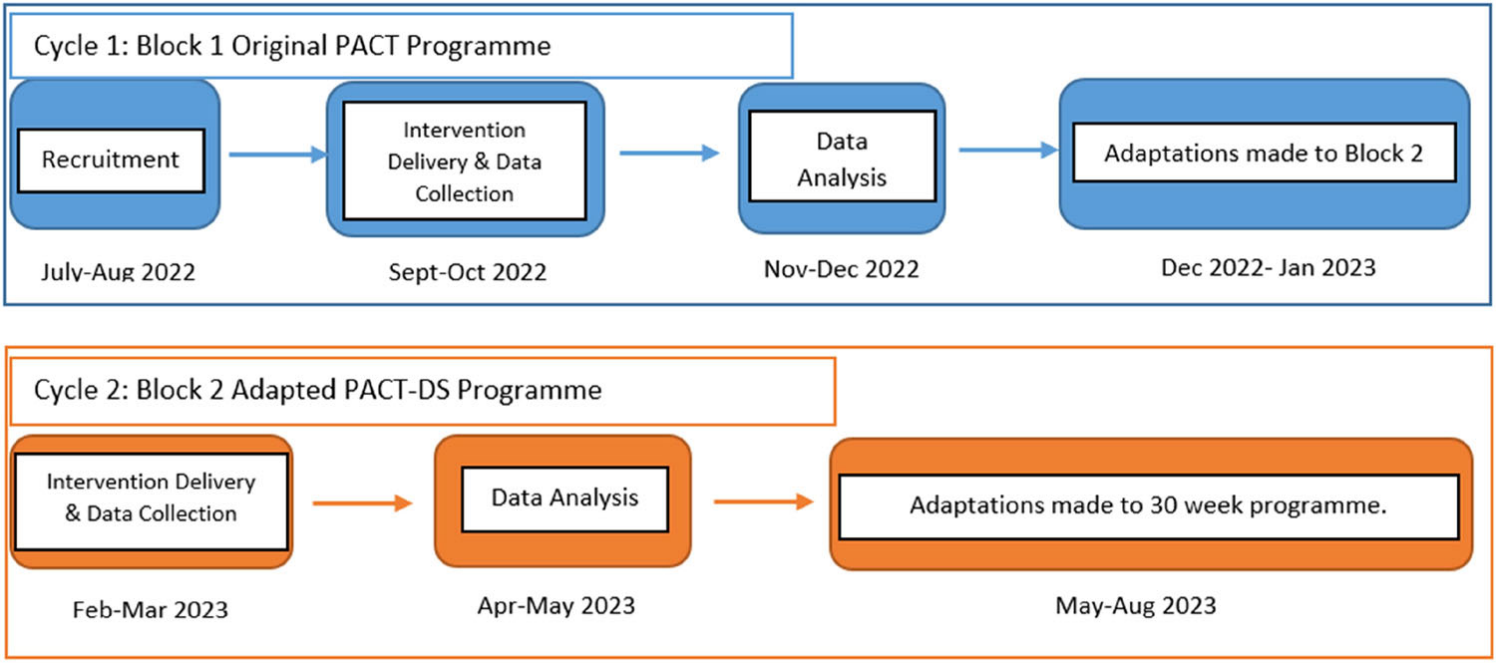
Cyclical process of programme adaptation.

Whilst some adaptations to the PACT programme were anticipated based on knowledge of the Down syndrome phenotype, the study aimed to learn directly from participant experiences; as such this study applied a CBPR framework, a collaborative approach that engages the community as coresearchers and thereby harnesses the strengths of researchers and community partners (Israel et al. 2005), and partnered with families from the Down syndrome community in the research process. Guided by DBR, an iterative process was used to adapt the PACT programme (Burgoyne et al. 2018) (see Figure 1). DBR is characterised by iterative cycles of design, implementation, analysis and re‐design to produce and advance knowledge and improve outcomes, and is increasingly used by researchers internationally in the field of education (Tinoca et al. 2022) as well as health research (Meyers et al. 2018).

In Cycle 1, parents were trained to deliver a 5‐week Block of the original PACT intervention (the original PACT intervention has 6 blocks, each lasting 5 weeks). Data was collected throughout delivery and used to inform understanding of families’ experiences and identify adaptations. Adaptations were then made to a second Block of materials, 4 weeks of which were delivered by parents in Cycle 2, with data collected throughout delivery (parents were not asked to deliver the consolidation week (week 5) in Block 2, as consolidation activities were essentially unchanged).

Prior to each Cycle, parents were invited to attend in‐person group training (approx. 2 h) to enable delivery. Training was delivered by two members of the research team KB and KH, a psychologist and a speech and language therapist. Training covered the background to the project, its aims and design, but focused on programme content and materials and examples of how to use the resources effectively. Parents were also given information on how to record programme delivery (see below). Following training delivery, parents were sent a recording of the training and a video recording of an adult delivering a PACT session to a (typically‐developing) pre‐school child.

Training was attended by all mothers in Cycle 1, either alone (*n *= 3) or with another family member (father *n *= 1; grandmother *n *= 1; adult sibling *n *= 1). In Cycle 2, four mothers (and one adult sibling) attended group training, with remaining mothers receiving 1:1 training online (*n *= 1) or in person (*n *= 1). Parents were given intervention materials at training and asked to deliver the programme every day (5 days a week) for approximately 20 min a day. Telephone and email support was available as needed from the research team throughout delivery. Individualised support was provided through observations of delivery (see below).

### 2.3 Measures

#### 2.3.1 Fidelity Measures

*Record forms*: Families completed a daily record form, electronic or paper depending on parent preference (25 sessions Cycle 1; 20 sessions Cycle 2), indicating time spent on the programme and completed components, and a weekly record form (5 weeks Cycle 1; 4 weeks Cycle 2) to record enjoyment, active involvement, and level/types support provided by parents each week.

*Observations*: Observations of intervention delivery were completed live during home visits and from video recordings. Observations were used to monitor fidelity to intervention and provide individualised feedback and support to families; this feedback also shaped programme adaptations. As such, home visits were planned to take place in the first weeks of delivery (Week 2 or 3) of each Block; however, in practice, the timing of observations varied across families.

In each cycle, two members of the research team KB and KH conducted a home visit with each family to observe delivery, although it was not always possible to complete observations during these visits (due to child refusals): Four (of 6) observations were completed in home visits in Cycle 1, and 5/6 in Cycle 2. Each family was also asked to submit a video recording of a PACT session: 5 (of 6) video recordings for observation were submitted in Cycle 1 (though note for one parent, the video was extremely brief and therefore observation data from the home visit was combined with the observation data from the recording) and Cycle 2.

Parents were asked to deliver observed sessions as naturally as possible. Two researchers conducted each observation by completing either a structured or an unstructured observation. Structured observations comprised a checklist of observations of key components of the session and behaviours central to fidelity of the programme (e.g., ‘adult introduces special word’). The duration of each component and the overall session was also recorded (in minutes). Unstructured observations were freely written and focused on adherence to programme delivery and ways in which parents adapted delivery.

#### 2.3.2 Experience Measures

*Parent survey*: Parents completed an online survey at the end of Cycle 1 about their experiences delivering the programme.

*Focus groups*: At the end of each cycle, parents were invited to a 2‐h in‐person focus group. In Cycle 1, a semi‐structured discussion explored parents’ experiences of PACT and suggested adaptations, and structured activities collected feedback on intervention materials. For Cycle 2, parents discussed their experiences of delivering the adapted intervention (PACT‐DS) and the data collection methods. See Supplementary Information S1 for topic guides. Focus groups were conducted by KB and KH and were audio‐recorded and transcribed verbatim.

#### 2.3.3 Analytical Strategy

We used a mixed methods approach to evaluate fidelity and experiences and identify adaptations. Quantitative data from the record forms, observations and surveys are reported descriptively. Focus group transcripts were analysed using Qualitative Content Analysis (Graneheim and Lundman 2004; Sugden et al. 2019): First, phrases, sentences or paragraphs with the same central meaning were labelled as meaning units. Inaudible/partly audible utterances where the meaning was not clear were not analysed. Meaning units were derived by putting together utterances containing aspects related to each other through their content and context, either from the same participant or across participants. These units were then shortened into condensed meaning units using words that closely approximated and accurately reflected the meaning units and then abstracted into codes (see example in Table 2). Mutually exclusive categories/subthemes were then created by grouping codes. Analysis was conducted by EP and VS (who were not present in focus groups) and confirmed by KB (present in focus groups). Finally, themes were developed to connect the meaning underlying the categories/subthemes through a process of consensus to ensure trustworthiness. A record of all changes made following each stage of analysis was kept for the purposes of increasing the credibility of the data. Data from Cycle 1 focus group were analysed separately: (1) for experience of PACT and (2) adaptations, due to the different nature of the topics.

**TABLE 2:** Example meaning units, code and category (Cycle 1 focus group).

Meaning unit	Condensed meaning unit	Code	Category/Subtheme
Yeah because that's what Child 2 tried to do, and I was like ‘No we aren't doing that, it's a different thing’, but he wanted to, because that's where his level is I suppose, that's what he's comfortable with.	Child wanted to do the familiar activities at their level rather than new activities	Familiarity impacts engagement	Engagement

## 3 Results

Cycle 1 results are presented and discussed first, followed by results and discussion from Cycle 2.

### 3.1 Cycle 1 Results

#### 3.1.1 Fidelity Measures

*Daily Record forms*: Across the six participating families, 125/150 daily record forms were received (83% return rate). Families reported completing 15–25 sessions over 5 weeks (*M* = 19.83, SD = 4.49) with individual sessions lasting 15–20 min (55%) or 21–25 min (28%) (see Supplementary Information S2, Figure S2.1). In most sessions (80%), parents completed all components (see Table 3).

**TABLE 3:** Cycle 1: Parent‐reported completion of intervention components.

Component	Frequency completed (% of sessions)	Frequency not completed (% of sessions)
Introduction	114 (91%)	11 (9%)
Reading together	117 (94%)	8 (6%)
Vocabulary	117 (94%)	8 (6%)
Stories	112 (90%)	13 (10%)
Reward	116 (93%)	9 (7%)

*Observations (n = 8)*: Observed sessions generally included all components (see Table 4). The time spent on delivering each component was broadly in line with programme guidance, though in some sessions, families spent longer on Reading Together and Vocabulary than on Stories.

**TABLE 4:** Cycle 1: Observed completion of intervention components.

Component	Frequency observed (% of sessions)	Time taken (% of observed sessions)
Introduction	6 (75%)	Less than 1 min (67%); 1–2 min (33%)
Reading Together	7 (88%)	4–6 min (43%); 7 or more min (43%); less than 4 min (14%)
Vocabulary	7 (88%)	4–6 min (57%); 7 or more min (43%)
Stories	6 (75%)	Less than 4 min (67%); 4–6 min (33%)
Reward	5 (63%)	Less than 2 min (80%); 2–4 min (20%)

#### 3.1.2 Experience Measures

*Weekly Record forms*: Across the 6 families, 23/30 weekly record forms were received (77% return rate). Reported levels of enjoyment and child active engagement were generally high (see Table 5).

**TABLE 5:** Cycle 1 weekly record form data: Enjoyment and active participation.

	Yes (% of sessions)	Mostly (% of sessions)	Some (% of sessions)	No (% of sessions)
Did your child enjoy the session?	7 (31%)	15 (65%)	1 (4%)	0 (0%)
Did you enjoy the session?	12 (52%)	10 (44%)	1 (4%)	0 (0%)
	**Yes** **(% of sessions)**	**Yes, with a bit of support** **(% of sessions)**	**Yes, with lots of support** **(% of sessions)**	**No** **(% of sessions)**
Was your child actively involved in the session?	2 (9%)	11 (48%)	10 (43%)	0 (0%)

Parents reported using various supports, including verbal prompts (96% of sessions), pointing (96%), signing (87%), rephrasing (70%), repetition (83%), slowing down (65%), additional pictures (22%), and a visual timetable (4%). Other methods included using objects (e.g., small toys) (13%), funny voices (9%), and extended wait time (13%).

*Parent survey (n = 5)*: All families reported the training was ‘About Right’ and felt ‘somewhat confident’ about delivering the programme following training. All families reported spending a maximum of 10 min preparing programme materials for each session, organising resources and gathering household objects to support activities. Three families (60%) reported that this became easier the more familiar they became with the programme (‘Once done the first week knew what to do’). Sessions were delivered by parents only (80%) or parents and an older adult sibling (20%). Four families occasionally divided PACT activities into more than one sitting. Most families (60%) delivered the programme during the day (9:00 AM to 3:00 PM), 1 family in the early morning (pre‐9:00 AM), and 1 family in the evening (6:00 PM onwards). Families reported it was ‘somewhat easy’ (60%) or ‘neither easy nor difficult’ (40%) to fit PACT into daily. Three families used other materials to support delivery, for example, farm animals and role‐play toys. Reported challenges were finding time in the day to carry out PACT, which was occasionally an issue (3 families); and children finding PACT uninteresting or too difficult (2 families) and difficult to concentrate on (4 families). Three families reported missing some parts of PACT that ‘were too difficult’. Parents reported enjoying the dedicated time spent together with their child. One parent shared that the programme ‘lifted a weight’ from their shoulders as their child had resisted being read to since starting school, but had got back to ‘loving books’ through the programme. Another parent reported that the programme facilitated their child's understanding and ability to follow instructions.

*Focus group (n = 4)*: As a warm‐up activity, parents were asked what words came to mind when they heard PACT‐DS: responses suggested parents saw the programme as engaging and supporting learning, for example, ‘fun’, ‘quality time’, and ‘shared learning’ (see Supplementary Information S3). Parent experience of PACT and adaptations were discussed separately in the focus group, and the data were analysed separately, due to differences in the nature of the topics and the activities used to collect the data.

Data related to parental experience of PACT were analysed into six themes (see Table 6): facilitators and barriers to child engagement, parental delivery of PACT, parental perspectives through experience of PACT, individual differences, the intervention itself and capturing intervention effects.

**TABLE 6:** Focus Group 1 – Experience of PACT (unadapted) themes and categories.

Theme	Categories
Barriers and facilitators to child engagement	Engagement Experiencing success
Parental delivery of PACT	Competing demands
	Flexibility
	Parent delivery of intervention
	Use of additional resources or visual supports
Parental perspectives through experience of PACT	Parent experience Parent priorities Parental expectations of abilities Observing progress


Individual differences	Child factors affecting delivery
	Child language levels
The PACT intervention	Contents of PACT
	Parent training
	Individual adaptations
	Repetition and consolidation over time
	Repetition of activities
Capturing intervention effects	Capturing change

#### 3.1.3 Parent Experience of PACT

*Facilitators and barriers to child engagement*: This theme included the categories ‘engagement’ and ‘experiencing success’. Parents discussed familiarity with the books, relating stories to personal experience, and external distractions: ‘It was a lot more hard work for me when she, when she didn't know the book… it didn't grab her interest as quickly so you didn't fit as much in a session’ (P1). Parents also spoke about the importance of the child experiencing success: ‘They've done something right and then they want to do something else don't they’ (P2).

*Parental delivery*: Parents talked about the impact on delivery of competing demands such as siblings and work: ‘My daughter is around then as well on those days, and then it is even harder cause even if I asked him question, she would be like, just butt in, before he can even give one anyway’ (P3). Flexibility in timing of delivery and expectations for each session were discussed: ‘Being able to change it, and also having the expectation that we might not finish…the section, the way I thought it was going to go, and that's OK’ (P1). Parents discussed a wide range of factors that affected delivery, including how they organised materials and prepared for sessions, the intensity of the intervention, confidence and use of techniques such as modelling, and the use of visual resources: ‘So I got my little folder and I ripped everything out first, and I had it cut up. It started alright, Week 1 and 2, and then it went all wrong. Because you just get busy, um but yeah’ (P2).

*Parental perspectives through experience of PACT*: Parents spoke about their experiences implementing the intervention, including positive experiences as well as feelings of worry and pressure: ‘so I do feel like I probably was doing it fast, to get them done, ‘cause trying to do it in the time, and not miss anything out….’ (P2). Parental priorities and wanting to maximise outcomes for their child were also discussed: ‘Speech at the minute is our main issue.’ (P2). Through delivering the intervention with their child, parents observed their child's abilities were at times beyond their expectations: ‘Yeah and some of the things that we did, she didn't say them or sign them, but showed an understanding that maybe I didn't know she had because she followed an instruction or maybe she showed me something’ (P1). Parents also spoke about observing progress: ‘after a few days he was starting to say ends of the chant…so that was obviously progress for me.’ (P4).

*Individual differences*: A wide range of child factors affecting delivery were discussed, including illness, tiredness, preferences and mood: ‘it depends what mood he's in. Sometimes … he would scooch right up to me and he'd be excited and other times he'd go “Oh no”.’ (P2). Parents also spoke about language abilities and use of sign: ‘some things he will only sign, some things he doesn't sign that he used to sign but he'll say it… its just very random.’ (P2).

*The PACT intervention*: Participants were positive about the training but also spoke about the importance of learning through doing, and the need for Down syndrome‐specific training resources: ‘It was quite hard to get, yeah because in the video obviously the girl can talk a lot.’ (P3). Parents discussed their own adaptations: ‘ I definitely adapted cause he didn't‐ he doesn't have that understanding for a lot of the game things.’(P2). Specific aspects, including the quantity of activities, the requirement to make resources and the importance of durability were also discussed: ‘So, I didn't want to not do anything with him…. To miss it out, but then….There's a lot to do…. In that little space of time’ (P2). Parents spoke about the impact of repetition for learning and engagement: ‘Each story, maybe doing some of the same exercises, would work. Cause then…you're using your time each time to teach them the new activity, you could actually get them straight into, what the purpose is, to learn more vocab.’ (P1).

*Capturing intervention effects*: Parents spoke about the potential limitations of measures in reflecting their child's progress: ‘Capturing progress and stuff…so when you get to the end and you're like, oh I know he's made progress but it doesn't look like he has with these ones and zeros.’ (P4). Parents again spoke about their child's abilities exceeding expectations: ‘And that was nice to see because she could show me what she knew, that I didn't know she knew.’ (P1).

#### 3.1.4 Adaptations

Data related to programme adaptations were analysed into four themes (see Table 7): principles to support learning, individualisation is key, adaptations to PACT structure, the unique role of parents as implementors (see Supplementary Information S4a and 4b for full details of categories and themes with examples of corresponding meaning units).

**TABLE 7:** Focus Group 1 – Adaptations themes and categories.

Theme	Categories
Principles to support learning	Language and learning opportunities Relatable content Repetition The importance of active involvement Use of text Visual resources Visual supports
Individualisation is key	Adaptation to the individual
Adaptations to PACT‐DS structure	Book choices
	Vocabulary
	Sequencing
	Existing activities that work PACT resources
Recognising the unique role of parents as implementors	Flexibility Parent delivery
	Generalising from PACT to other learning opportunities Parent training Parent perceptions of child abilities

*Principles to support learning*: Over‐arching principles to support learning were discussed included repeated exposures to language, relating intervention to the child's personal experience, active involvement and the importance of repetition:

‘Yeah, I think it's about exposing them to things… I was trying to teach her makaton and I was doing the signs over and over again and she just wouldn't do anything back. And then 1 day, she just signed ‘more’ and it was like a lightbulb moment … you're exposing them to it and… it's gonna come out, whenever, in their own time’ (P1).

Visuals, including objects, photographs, symbols, manual signs and text were important to all parents: ‘I think the photographs have been great… he was saying ‘bird’ the other day to the parrots and then as soon as he saw the photo with the parrot with the word Parrot.’ (P4).

*Individualisation is key*: Parents spoke about heterogeneity across children and the need for flexibility so parents can tailor the intervention to their child, for example, by providing core and optional activities and suggestions to make activities simpler or more complex: ‘ if your child's not got it, what're you gonna do to take it a step back? So like a step forward and a step back…’ (P1).

*Adaptations to PACT‐DS structure*: Parents spoke about all three core elements of PACT‐DS (shared book reading, vocabulary and sequencing) and discussed features that worked well and suggestions for important considerations for adapting the programme to be more accessible for children with Down syndrome: ‘I think they're good, I think what you need is like two….Two nouns that are very much “this” and “this” that they can grasp, but then other, more, like verbs. Because for – our children, like prepositions, they're not, well they probably will know them, when they begin to talk, they don't use prepositions.’ (P1). Parents spoke about particular challenges with more abstract vocabulary and sequencing activities: ‘Child4 doesn't really get the order, he‐he was able to say a sentence‐ish for each one, sometimes it was just….Walking on head, that's what he said you know for‐for how they're walking on head [laughs]. And I was like‐ so then I made it into something but he‐ he would have no idea how‐ like middle? No.’ (P4). Participants also discussed the resources provided to deliver PACT: ‘I do use them, or I do adapt them. and I think sometimes it's useful, sometimes it's not, sometimes you had to adapt it a lot sometimes you just have to tweak it or you have to repeat a bit. But it's good, it's good.’ (P4).

*Recognising the unique role of parents as implementors*: Factors that enabled parents to deliver PACT effectively were identified, including capacity for flexible delivery, tailoring to the child's interests and motivations, and opportunities to extend PACT to other contexts: ‘…this whole programme opens doors for other books that you could use with them, that are more specific to your child.’ (P1). Parent perceptions of their child's abilities and how that influences their capacity to engage with the programme were discussed: ‘They don't have them concepts, do they? *…*.some parts are really good aren't they? And other parts are a bit like ’ugh!’… they just haven't got them at all.’ (P4). Finally, parents discussed aspects they thought needed highlighting in future training: ‘But also it would have to be really stressed in there, like, you don't have to do this bit.’ (P4).

#### 3.1.5 Cycle 1: Discussion

The aim of Cycle 1 was to pilot 5 weeks of the PACT programme to examine family experiences and identify adaptations. Whilst families’ experiences varied, it is clear there are broad patterns which illustrate strengths of the original programme as well as changes that would enhance feasibility. Data indicates high levels of adherence to the procedure (Durlak and DuPre 2008), implying that the core components of the programme are broadly acceptable and feasible for families. Parents and children generally enjoyed the sessions, though parents also expressed some anxieties about fitting in all components of the programme, and at the right pace.

We did not observe fidelity to all components as frequently as parents reported completing them, though fidelity was still high in observed sessions. This is likely due, at least in part, to factors associated with being observed; for example, some children were distracted by the researcher/camera, and for parents, this was often the first time they had been observed which may have led them to shorten or omit components during observations. Families reported skipping over and/or adapting aspects of the programme that were too difficult for their child: On some occasions families did ‘skip’ activities which they felt were too difficult; more typically, they adapted them so they were more suitable for their child. Flexibility to tailor the programme to children's strengths and weaknesses is therefore important to maintain fidelity. Stories was clearly the most challenging component of the programme: this component was most frequently omitted, and often afforded less time than the other core components. This was not surprising given that children with Down syndrome face significant challenges in storytelling (Chapman and Hesketh 2000) but did highlight this component as particularly in need of adaptation.

Children were actively involved in the sessions. Active participation is a key aspect of the intervention, and parents excelled at engaging their children in the activities using various methods, highlighting the unique position of parents in supporting their children's language learning. Parents noted that experiencing success was key for engagement, but external distractions and lack of familiarity with the book made it challenging to maintain children's interest. This finding highlights the importance of pitching intervention activities at the right level for children to ensure they experience success and maintain motivation (Gilmore and Cuskelly, 2014).

Data collected in Cycle 1 led to the following adaptations to the PACT programme (See Supplementary Information S5 for further details): (1) *Enhanced repetition and consolidation* (e.g., increased opportunities to repeat and consolidate target words and activities); (2) *Reduced time pressure* (removing introduction and reward as specific components and exact timings for components); (3) *Tailoring to individual needs* (e.g., suggestions for step‐up and step‐down options for activities), (4) *Smaller steps for learning* (teaching fewer target words and extending support for challenging activities), (5) *Supporting engagement* (e.g., optional visual timetable), and (6) *Increasing visual support* (through photographs and Makaton signs). See Table 8 for an overview of the adapted programme.

**TABLE 8:** Overview of adapted PACT‐DS language session.

Component	Description
**Reading together**	Read books together and talk about the story
**Words**	Learn about new words and their meanings
**Using words**	Ordering, describing, and retelling stories

*Note*: Specified timings were removed as part of the programme adaptations, but parents were advised to spend approximately 20 min in total delivering the session with roughly the same amount of time dedicated to each component.

### 3.2 Cycle 2 Results

#### 3.2.1 Fidelity Measures

*Daily Record forms*: Daily record forms were adapted for Cycle 2 in line with adaptations to the programme, resulting in a maximum of 20 sessions recorded per family over 5 weeks of teaching. We received 115/120 daily record forms (96% return rate). Families reported completing 13–20 sessions over 5 weeks (*M* = 18.33, *SD* = 2.88) and delivered most sessions (53%) in 15–20 min or 21–25 min (29%) (see Figure S6.1 in Supplementary Information S6). In most sessions (96%), families completed all components (see Table 9).

**TABLE 9:** Cycle 2: Parent‐reported completion of intervention components.

	CYCLE 2	
**Component**	Frequency completed (% of sessions)	Frequency **not** completed (% of sessions)
**Reading together**	105 (91%)	10 (9%)
**Vocabulary**	108 (94%)	7 (6%)
**Stories**	104 (90%)	11 (10%)

*Observations (n = 9)*: Data collected from observations of intervention delivery in Cycle 2 are presented in Table 10.

**TABLE 10:** Cycle 2: Observed completion of intervention components.

	CYCLE 2		
**Component**	Frequency observed (% of sessions)	Frequency **not** observed (% of sessions)	Time taken (% of observed sessions)
**Reading together**	8 (89%)	1 (11%)	4–6 min (50%); 7 or more min (50%)
**Vocabulary**	8 (89%)	1 (11%)	Less than 4 min (25%); 4–6 min (50%); 7 or more min (25%)
**Stories**	6 (67%)	3 (33%)	4–6 min (67%); 7 or more min (33%)

**TABLE 11:** Cycle 2 weekly record form data.

	Yes (% of sessions)	Mostly (% of sessions)	Some (% of sessions)	No (% of sessions
**Did your child enjoy the session?**	7 (32%)	13 (59%)	2 (9%)	0 (0%)
**Did you enjoy the session?**	9 (41%)	11 (50%)	2 (9%)	0 (0%)
	**Yes**	**Yes, with a bit of support**	**Yes, with lots of support**	**No**
**Was your child actively involved in the session?**	11 (50%)	6 (27%)	5 (23%)	0 (0%)

#### 3.2.2 Experience Measures

*Weekly Record forms*: We received 22/24 weekly record forms (92% return rate). Reported enjoyment in the sessions was high, as was children's active involvement (see Table 11). Parents reported using a range of strategies to support their child's participation, including verbal prompts (100% of sessions), pointing (95%), signing (95%), rephrasing (55%), repetition (91%), slowing down (41%), additional pictures (14%), visual timetable (27%). Additional methods included using objects (e.g., small toys) (9%), funny voices (9%), and extended wait time (13%).

*Focus group (n = 3)*: Data were analysed into three overarching themes: Parental views on revised programme (PACT‐DS), Further adaptations to PACT‐DS, and Barriers and facilitators of successful implementation of PACT‐DS. Full details of the themes, categories/subthemes and codes with meaning unit examples are available in Supplementary Information S7.

*Parental views of the revised (PACT‐DS) programme* were generally positive while acknowledging challenges. There was agreement that the revised programme improved upon the original programme for several reasons including increased flexibility allowing tailoring to individual children, inclusion of Makaton, repetition of activities and slower pacing, for example: ‘because a lot of the, um, ways of doing like the activities were the same so I wasn't teaching her how to do a new activity, it was just the learning of the words and the content’ (P3). Parents talked positively about the experience of success with children often exceeding parental expectations in what they were able to learn.

‘The Using Words bit, initially I just thought…I don't know if he's going to get this…but then he really surprised me….I [was] just like “Oh wow” cause I…wasn't, yeah, totally confident on modelling it but then we sort of just went through and he seemed to understand’ (P1).

Parents enjoyed using recommended step‐up or step‐down tasks to reflect different levels of ability and the heterogeneity of children with DS. ‘I didn't ever once look at the extra challenge bit and go ’Oh he's not going to be able to do that’ and feel bad about it…. Cause‐cause you also had one to make it easier’ (P1).

Parents also identified some challenges, such as teaching challenging concepts as well as commenting on the effort and preparation time required: ‘Where we struggled was where you had, like a picture of a lady, just a random lady, and have mum under it, so I'm thinking that's not his mum, that's not any mum he's ever known, that's just a lady, you know. ’ (P1) ‘There was a lot of pre‐preparation involved in this wasn't there? It was kind of like, I couldn't just open it and do it, I had to like, the night before, I had to – that's a lot, you know that's a lot of effort for me’ (P1)

*Further adaptations* to PACT‐DS featured as a major theme. Parents provided suggestions to the intervention materials to further enhance accessibility and effectiveness for their children's learning. These suggestions included targeting fewer concepts and focusing on specific concepts to target: ‘I think having those going over longer, less concepts to learn, less words to learn, so you're repeating them.’ (P1); ‘Over and under, I mean that's brilliant…So, yeah, more prepositions’ (P1).

Parents also felt it was important to provide opportunities to relate words learnt to the real world: *‘*So you've got more chance to, um, show them in the real world*’* (P1), and to have practical hands‐on activities to keep children engaged: ‘Some sort of like pegs on a washing line. Because I could put it, you know that way of sort of teaching where you make a mistake on purpose’ (P3). Parents also emphasised the importance of how the materials are presented: ‘I think what made it easier in the original one is sort of, the colour‐coded sections, cause when you've just got a photocopied sheet in black and white to read, and you're trying to skim read and you're feeling pressure.’ (P1)

Flexibility in timing of delivery was important so that it could fit around family schedules: ‘Allowing for a holiday week or two’ (P3); Cause some of these aren't, we aren't doing them over a week, are we?’ (P1).

*Barriers and facilitators of successful implementation*: Parents emphasised the importance of repetition, simplicity, and familiarity for child learning and engagement. ‘The repetition was great though, like [child's name] with the first, the other 2 books and by the end like he‐ he wasn't saying, trying to say over and under at the beginning or sign it and by the end he was doing over and under’ (P1) ‘With our children, you need them to‐ to know what they're doing so they need familiarity’ (P3)

Resources provided were identified as very important, especially the presentation of materials and visual supports including the use of pictures versus photographs: ‘I know, I know, but it's a mix isn't it, because the photographs do work well when you're trying to categorise, so with the adult and the child and you're putting them in piles, or you know another concept where you had in the original PACT, where it was field or farm, photographs work great there. It's just concepts, I think where they don't’ (P1). There was a lot of discussion on choice of books and how these needed to be relatable: ‘New House was really apt for us because we'd just moved house, so it was like a relatable life event’ (P1).

Parents expressed concern about the potential lack of diversity of parents who would agree to deliver this type of intervention due to time demands and other commitments. ‘You're going to end up with, um, data from families like us, you know, where the parents are very invested in their children, and that's the only thing. I think you'll probably get a lot of dropouts from possibly families that haven't got the capacity to do it’ (P1)

#### 3.2.3 Cycle 2: Discussion

Fidelity increased in Cycle 2 (96% of sessions with all components complete), suggesting that the adapted programme may have increased adherence. Encouragingly, children were actively involved with less support, which indicates that the adaptations enhanced children's ability to actively participate in the sessions. Narrative (Stories) continued to be the most challenging aspect of the programme, as this remained the most missed component in record forms and observations. Nonetheless, feedback from the focus groups suggests parents were pleasantly surprised by what their child could do in the activities in this component. Fatigue may play a role, as Stories occur at the end of the session. Overall, parents reported positive experiences of the adapted programme, while acknowledging some challenges that likely would apply to other interventions (e.g., visualising challenging concepts and preparation time) and suggesting further adaptations that could be made (see Supplementary Information S5 for programme adaptations).

## 4 General Discussion

The aim of this exploratory study was to work collaboratively with families to pilot and adapt a parent‐delivered language intervention for children with Down syndrome. Our approach was to take an evidence‐based programme (PACT) that had been shown to be effective for other populations (Burgoyne et al. 2018) as a starting point. Data collected in Cycle 1 suggest this was an effective approach: Record forms and observations indicated that parents were able to deliver the programme with high levels of fidelity, with children actively engaged in the activities, and parents reporting high levels of enjoyment. It was clear that many aspects of the original programme worked well for this population and these were retained as a result.

We also anticipated adaptations would be needed to increase fidelity and enhance effectiveness; whilst some adaptations could be predicted based on knowledge of the Down syndrome phenotype (e.g. Fidler and Nadel 2007), our aim was to learn directly from participant experiences. Drawing on principles of CBPR (Israel et al. 2010, 2005) and DBR (Tinoca et al. 2022), this research employed iterative cycles of design, implementation, analysis, and re‐design to produce the adapted programme. This process identified a range of adaptations that were aligned with real‐world experiences. Fidelity improved with the adapted programme, suggesting that the changes to structure, content and materials increased adherence to the intervention activities. This was echoed by an increase in parent and child enjoyment and active involvement of the child with less need for support.

Families from the Down syndrome community were key partners throughout this research. This study illustrates that parents are uniquely positioned to tailor intervention to their individual child and provide daily support and frequent opportunities for reinforcement and generalisation of learning (Roberts et al. 2019; Walsh et al. 2024). It is important to note, however, that parents also emphasised the need for flexibility in delivery and given the intensity and duration of the PACT‐DS programme, coupled with parent concerns related to time commitments associated with parent‐delivered intervention (Walsh et al. 2024), it is important to conduct further research evaluation to understand whether intensive implementation is feasible over time. Furthermore, parents highlighted that delivering intervention to their child enhanced their own understanding of what their child was able to achieve, as they demonstrated abilities that were beyond their expectations. Similar to findings reported by Walsh et al. (2024), this implies that one of the benefits of parent‐delivered interventions may be in demonstrating to parents their child's learning potential, as well as providing children with new opportunities to learn. Further, parents provided critical learning and ideas about necessary adaptations that aligned with their experience. These relate both to the intervention content and to future research, including ways to assess progress and ensure representation in participant groups.

The next step in developing this work is a feasibility randomised controlled trial (RCT) to evaluate the feasibility of a definitive RCT and explore initial evidence of the programme's potential to support language and literacy development of children with Down syndrome (Burgoyne et al. 2023). The current study highlights the importance and value of working with families who have children with neurodevelopmental conditions to inform our understanding of how support can be tailored to their needs. Our approach shows promise, and we hope that this paper will serve as a foundational model for future research in this area. Further studies are now needed to assess and build upon our work, to advance the development and understanding of how best to support families.

## 5 Conclusion

This research highlights the critical role of parent involvement in language interventions and paves the way for further exploration in this area. By continuing to learn from families and refine our methods, we can better support the developmental needs of children with Down syndrome and other neurodevelopmental disorders.

## Conflicts of Interest

The authors declare no conflicting interests.

## Supporting information




**Supporting**: jlcd70139‐sup‐0001‐SuppMat.docx

## Data Availability

Data are available from the authors on reasonable request.
