# Supplementary material for: The Power of Partnership: Adapting Early Language Intervention for Children With Down Syndrome Through Family‐Researcher Collaboration
Source: Int J Lang Commun Disord. 2025 Oct 11;60(6):e70139. doi: 10.1111/1460-6984.70139 (PMC12515274; doi:10.1111/1460-6984.70139)
Supplement: Supplementary file 1 — Supporting: jlcd70139‐sup‐0001‐SuppMat.docx [file JLCD-60-0-s001.docx]

**S1: Focus group topic guides**

**Cycle 1 focus group**

***A: Warm up activity***

What words come to mind when you hear PACT-DS? (online software Mentimeter to generate a wordcloud)

**B: *Semi-structured* *focus group discussion***

***PART 1: Experience of PACT***

1. What was your experience of PACT?

1. How long did it take you to feel confident delivering the programme, and is there anything we can do, or add to the training, considering that?
   1. What were your thoughts in terms of what we included, what we didn’t include, the size of the group, where we had it..?

1. How best do we capture how children are making progress?

***PART 2: Adaptations to PACT for children with Down syndrome***

1. What changes would you like to see?
2. After trying all the materials is there anything that you'd want to keep?
3. Thinking about the overarching programme rather than individual activities, would you keep the structure of it as it is?

***C: Activity*** *(materials from Block 2 to review and write suggested adaptations on post-it notes)*

- How appropriate are the words?
- What are your thoughts on 5 words for each week?
- Is there anything you would change about the introduction? What are your feelings about the introduction?
- What're your feelings about reading together and are what we might want to change about that?
- What are your views on the bookmarks?
- How has one book a week worked?
- What do you think about the choice of books? Would you use different books?
- What would you change about the vocabulary bit?
- Would you want to keep the special word cards?
- How we might change some of these games?
- Are there about other games that would be good to build in?
- What do you think about the stories?
- Is there anything that you want to reflect on about week 5 and whether you'd keep that the same or change any of it?

***D: Activity*** *(present list of suggested adaptations based on cycle 1 and choose key adaptations)*

**Cycle 2 focus group**

**Topic 1 - What are your views on the adapted programme?** (30 mins)

**Follow up prompts:**

- What is working well?
- What is working less well?
- Is there anything else we could do to make it better for you and your child?

**Topic 2 - What are your views on how we have collected data?** (30 mins)

**Follow up prompts:**

- Are there ways we could adapt any of this to make it better?
- How did you feel about X – before and after?

**Summer Workshop – How would this work best**

**S2: Figure S2.1: Time spent on intervention delivery per session in Cycle 1.**

*
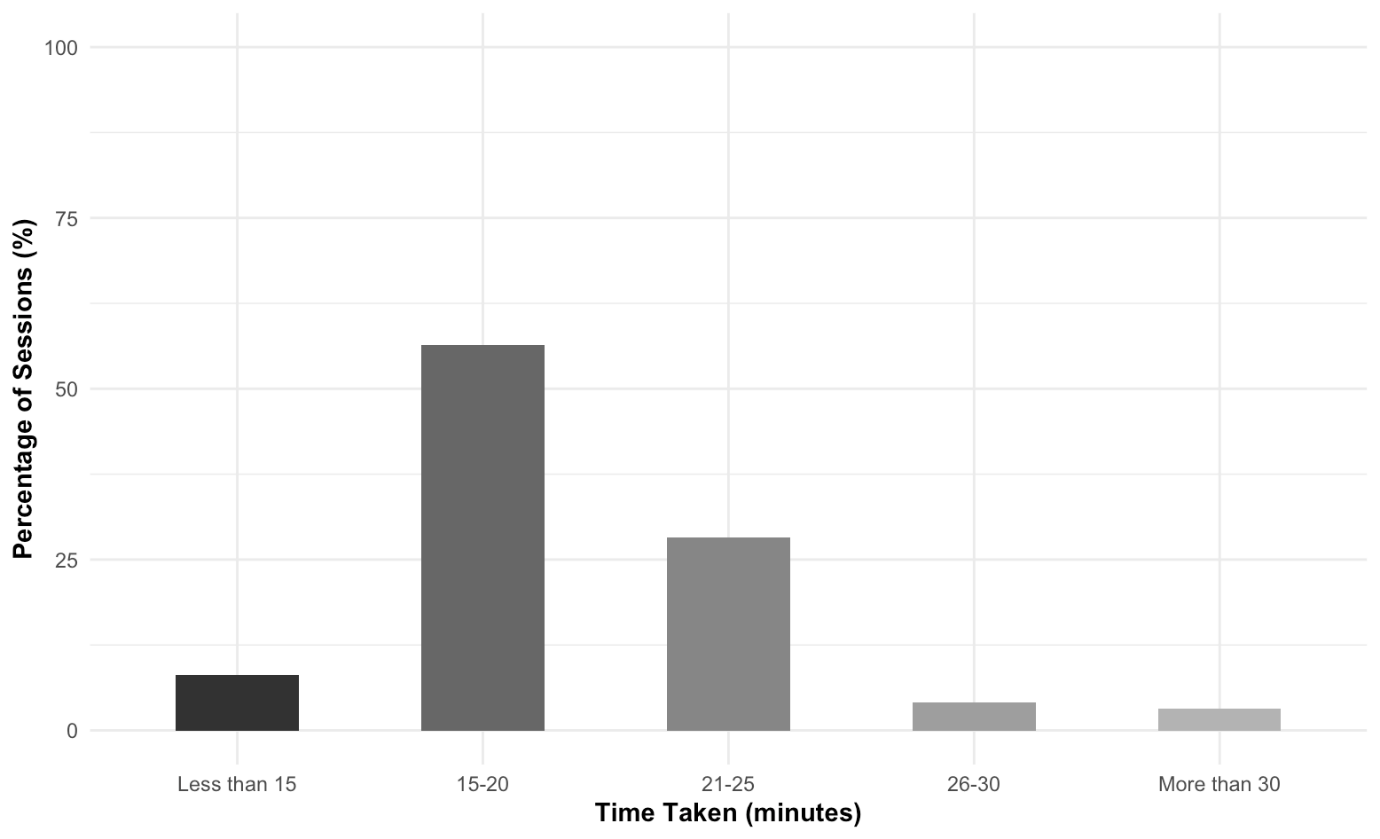
*

Figure S2.1: Time spent on intervention delivery per session in Cycle 1.

**S3 – Focus Group Word Cloud (Cycle 1)**

**
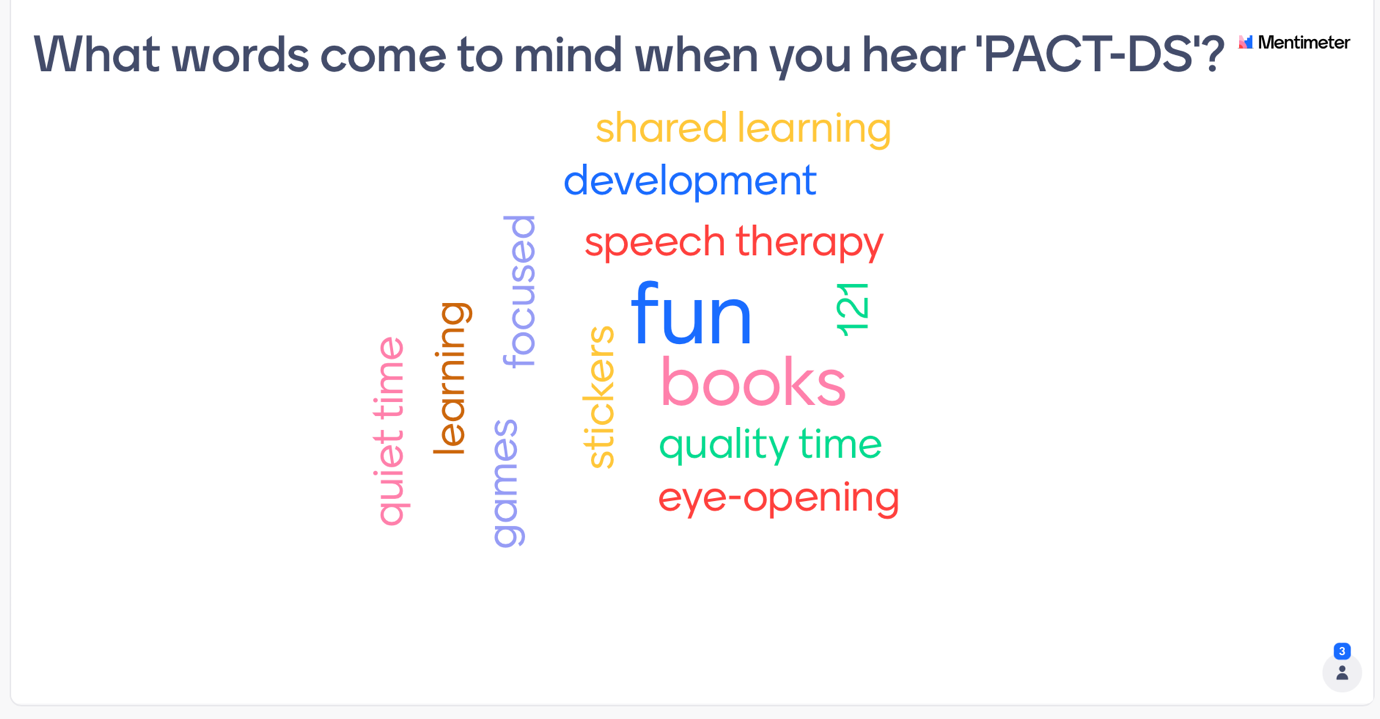
**

**S4 – Focus Group Analysis – Cycle 1**

**S4a: Experiences of PACT**

| **Category** | **Code** | **Meaning Unit Examples** |
| --- | --- | --- |
| Theme 1: Child engagement | | |
| Engagement | engagement over time | “See we started off well and then it all went a bit wrong, on the week 5 I was a like "Oh no", especially when Child2 chucks it all over the floor, I think he did it when you were there, I was just like "Oh no".” (P2) |
|  | external distractions impact engagement | “it was when outside things affected us that couldn’t help. Like when the neighbour was bringing a bin in and then she wanted to go and say hello, and all that sort of stuff. Or the dog barked at the postman, just little disturbances, that probably for any other child..It probably wouldn't be that much but for our children..[Bangs on table] "What is going on, I need to see!".” (P1) |
|  | extrinsic rewards | “It's bribery for me.You know, lolly, she said it when you were there.You, you can have a lolly if we do this, and that’s what normally works.” (P1) |
|  | familiarity impacts engagement | “It was a lot more hard work for me when she, when she didn't know the book, um. I don't- just cause it didn't grab her interest as quickly so you didn't fit as much in a session” (P1) |
|  | enjoyment of imitation | “she liked trying to copy the words back” (P1) |
|  | negative anticipation | “Mine will go "Oh no!"Yeah, well he still says it a lot” (P2) |
|  | parent-child time is special | “I think for me, she liked it more because it was mine and her time and um, I get a lot of time with her younger sister, um and obviously since she's come home we've not had as much one-to-one time with her.... So she enjoyed that. That is was her and mummy time.” (P1) |
|  | personal experience of story events | “Then with the last book, even though she didn’t know it, because it was about a pet dog, and we've got a pet dog, that helped. That, like, lived learning experience.” (P1) |
|  | positive anticipation | “now he- he gets excited now, when I say its time for PACT. He finds his chair” (P3) |
|  | active involvement-tactile cues | “she liked, um when we did it, I made her do it with her finger” (P1) |
| Experiencing success | success supports engagement | “Yeah, they get confident then. They've done something right and then they want to do something else don't they” (P2) |
|  | success with matching activities | “He really just wanted to match the pictures and he felt like he was doing well at that, cause he was linking it to the book.” (P2) |
| Theme 2: Parental delivery of PACT | | |
| Competing demands | occupying siblings during delivery | “if someone was coming to pick up my other daughter, or you know, to have her for an afternoon, i'd say "Could you stay an extra 20 minutes and just play with my other daughter while I do this?". Um, or my husband would look after her” (P1) |
|  | sibling involvement is a challenge | “My daughter is around then as well on those days, and then it is even harder cause even if I asked him question, she would be like, just but in, before he can even give one anyway” (P3) |
|  | siblings | “because you've got to be quite organised. Because we have a one year old as well, so..” (P1) |
|  | work commitments | “Um, with great difficulty. Um, working as well….. So, I work every day and so does my husband, so the only time we get to do it, is in the evening,” (P4) |
| Flexibility | adaptations in response to child state | “I did find that after dinner, he was tired in the afternoonish, like sometimes..I ask him less questions when he's tired because I feel like I'm going to push him a little too much [laughs]” (P3) |
|  | flexible expectations | “Being able to change it, and also having the expectation that we might not finish…the section, the way I thought it was going to go, and that's OK” (P1) |
|  | flexible scheduling around child | “so the only time we get to do it, is in the evening, when he doesn’t want to do it.we're making use of the weekends and doing it earlier, so we [laughs] we do it Saturday and Sunday.” (P4) |
|  | flexible scheduling around other commitments | “Yeah I've done, sometimes ive done 2 sessions, on a Saturday, one in the morning.... And one in the afternoon.Cause I've had to maybe catch up, or I've thought "Next week, we've got a busy week" (P1) |
| Parent delivery of intervention | attention monitoring | Yeah, sometimes at the begin- I'll know because I'll say something totally random, like maybe if it was Handa's Surprise, I'd say "Oh, it's a whale!" and then she'd look at me or sometimes she'd go "yeah" and then I'd know that she wasn’t understanding. But if she looked at me as if to say, "No?". Then I knew she was listening... Um, so yeah sometimes I'd do it wrong on purpose, to see if she noticed. And sometimes she did, sometimes she didn't.” (P1) |
|  | becoming more flexible about delivery | “I think being a teacher myself, it's like no we are doing it, ive planned this, its going to go this way, and then I've thought no. For both of us, we will just change it as we go along and see.” (P1) |
|  | communication opportunities | “I was letting him finish it off and he was doing it” (P4) |
|  | confidence increased with practice | “I think, Week 1, I was alright after Week 1. After I had done a whole run of- of..” (P4) |
|  | intensity of intervention delivery | “Or even having like, say its a 6- well not 6 week, but this is when we are going back, and if we do it all before we get back, and then you can try and do it in your own time.” (P2) |
|  | organisation of materials | “I'm far less organised.... I'm a bit like, just 10 minutes or something..You know, we're gonna do it now, rip rip rip quick and sort of try and, yeah, far less organised.” (P4) |
|  | parent enjoyment motivates child | “I just start it without him. Cause he just goes in the corner and try and distract himself with something else. So I'll be like "Oh, duh duh" and then, he- after, because that works with him anyway... Like distraction. So, if he isnt doing sommet and you want him to do it. If I pick my phone up and look really interested in something, or a book, then he's too nosey, he's like, within a minute, he's like "What're you doing?"He gets into it then, so.. You've just got to like, look like you're enjoying it without him.... Cause he feels like he's missing out.” (P2) |
|  | parent used modelling | “I made the sentence up.... Because she wouldn't do it.” (P1) |
|  | parental motivation | “Like I find I wanted to get through it all.” (P2) |
|  | parental struggle | “So it's, it's difficult really.. Um.Yeah, I've really strugg-, I-I didn't think I would but I've really- I've not struggled with doing it, once you know.” (P4) |
|  | preparation | “So I got my little folder and I ripped everything out first, and I had it cut up. It started alright, Week 1 and 2, and then it went all wrong. Because you just get busy, um but yeah” (P2). |
|  | siblings engaging in intervention | “I've got a older girl and she got jealous and yeah, she wants me to do PACT again with her.... Even though it's easy for her..But she still quite likes it, she likes all the.. Yeah..” (P3) |
| Use of additional resources or visual supports | pictures to support understanding | “I think as well, that's where the cards come in though, so you can, rather than yes or no, you can make it more open ended by saying, um, which food does the caterpillar eat first and you've got all of them… but you can, if you're using the manipulatives to support the question. Because they can't- because they can't talk, you give them another option to, to show it.” (P1) |
|  | using objects supports engagement | “So I have had to sort of get a load of objects together, and make it a bit more [laughs] fun...To get his engagement” (P3) |
| Theme 3: parental perspectives through experience of PACT | | |
| Parent experience | fun | “generally I think, you know, it's quite fun” (P3) |
|  | parental worries about delivering PACT | “That's the thing, because I was worried that I had let you down or something. Not that you put that but as soon as I emailed and you were, yeah, I didn't, I felt alright then” (P3) |
|  | positive parental attitude | “I feel it's positive.” (P1) |
|  | pressure to meet schedule | “so I do feel like I probably was doing it fast, to get them done, cause trying to do it in the time, and not miss anything out....” (P2) |
|  | using questions | “I did get told by a speech and language therapist to not ask too many questions so it was quite nice to ask some questions” (P3) |
| Parent priorities | focus on child speech | “Speech at the minute is our main issue. He's frustrated, and I'm frustrated.” (P2) |
|  | maximise learning | “cause you want him to learn as much as possible.” (P2) |
| Parental expectations of abilities | unexpected abilities | “Yeah and some of the things that we did, she didn’t say them or sign them, but showed an understanding that maybe I didn’t know she had because she followed an instruction or maybe she showed me something……like how she manipulated things, so yeah.” (P1) |
| Observing progress | linking progress with intervention | “Like, but I noticed these things this week, um.. That maybe she's showed that, um, that she's, um, got an understanding of, um, im trying to think of an example but I can't. Because, some things as well I've thought, "How does she know that?" and I thought she must of either learnt it at one of her other groups or at nursery or something.. But it's then hard to qualify then, is that, has she learnt it in the programme or has she learnt it elsewhere and that's the difficulty isnt it?” (P1) |
|  | observed progress | “I think like, when we were doing The Gingerbread Man, um after a few days he was starting to say ends of the chant, you know the song, and that was, so that was obviously progress for me. He had learnt that …” (P4) |
| Theme 4: Repetition and consolidation are key | | |
| Repetition and consolidation over time | repetition, consolidation and smaller steps in learning | “make them like not week but make them like longer phases, so you can keep revisiting and revisiting them. I know that is what the purpose of day 5 is, but I feel like more of it, more repetition. I don't know how you can do it but…” (P4) |
| Repetition of activities | repetition impacts engagement | “I just want to say the need more repetition, I think sometimes we do a game and you're like "Oh, I could do with doing this another few times and it's gone. Like, you know, normally I repeat that and repeat that. Cause normally he likes, he, like one of you said, he does like repetition... Not all in one day but he will "Oh, yeah! I've done that before, I like that because I've done it before". (P4) |
|  | repetition maximises learning | “Each story, maybe doing some of the same exercises, would work. Cause then you're not- you're not, its not wasting your time but you're using your time each time to teach them the new acitvity, you could actually get them straight into, what the purpose is, to learn more vocab.” (P1) |
|  | repetition of activities | “But also, maybe repeat the activities with the, the same acitvities...With each book..So, maybe on day 1, it’s the matching the pictures from the story with the- in some way. Um, and so then the, topic changes, so the vocabulary changes, but actually doing..Yeah the process is the same, so.. You aren't fighting with them.. Yeah because you do the same thing with the beginning, the middle and the end, each..” (P1) |
|  | repetition of books | “But, then she enjoyed looking at it and we read it together as, that was the story. But again, she sounds very different in that she takes comfort from reading the same books over and over again.” (P1) |
| Theme 5: The impact of child factors | | |
| Child factors affecting delivery | child mood impacts engagement | “it depends what mood he's in. Sometimes he'd see the, the book and like everything there and I'd go "right, ready?" and he would scooch right up to me and he'd be excited and other times he'd go "Oh no". And I'm like "Yeah, we're doing it again!" (P2) |
|  | child preferences | “If I say should we read Handa's Surprise again today, Child4 will go "No"."I want to read something else". He won't- he won't say that but he'll go "That one" instead, you know. He wont- he wont want to do it. I did think about that.” (P4) |
|  | child refusal | Child2 is the opposite, its always me and him, so he like "oh no, not her again" "She wants me to do some learning again" (P2) |
|  | finding time when child isn't tired | “Yeah, if he is really tired definitely, although he kind of, on nursery days. But, he was too tired anyway,...On days he wasn’t in nursery I did it in the morning, cause he's a bit better...., but, on nursery days there no chance in the mornings, .. Do it just before dinner but…” (P3) |
|  | following adult direction | “it's- it's just different than having a typical child, who will just do it and sit there and.. Not all typical, I don’t have any typical children so I-I just asume that they're all amazing. And you know that they all just do what they're told, but um, may- maybe not.” (P4) |
|  | impact of illness | “Also, at the moment, I'd say 50% of the time he is ill. He's off school again today, um. There's no point doing it when he is ill.” (P4) |
| Child language levels | communication attempts at different levels | “But, I know he got the book and when he saw the pictures of running he started running round the room so I knew that that's what he knew that that's where he legs it so I knew that he got it and he likes it, but to say what happen- even just to get 3 - a 3 word sentence you aren't gonna get it.” (P2) |
|  | impact of comprehension | “but when I started asking him some of the questions.... I just- I could tell he just didn't know what I meant, for the game side of it” (P2) |
|  | tailoring to the right level | “Some of them are too abstract aren’t they?” (P4) |
|  | variability in use of means of communication | “some things he will only sign, some things he doesn’t sign that he used to sign but he'll say it, and there’s no rhyme- there’s no reason, its not like its every word beginning with a certain thing, its just very random.” (P2) |
| Theme 6: The intervention itself | | |
| Contents of PACT | durability | “she's a bit of a wrecker, like, so it was a bit too flimsy, so as soon as she went through the first one and it ripped she was "Oh! Oh!" and then she did it on purpose with the second one - "OH!" (P1) |
|  | making resources | “There was no perforations so you were there like.. For gods sake! I’ve got to cut all these little bits out! It was a bit irritating.” (P4) |
|  | quantity of content | “So, I didn't want to not do anything with him.... To miss it out, but then.. There's a lot to do.... In that little space of time.... Cause sometimes there a lot isn't there?” (P2) |
| Parent training | learning through doing | “I think you have to do it don’t you?” (P3) |
|  | training was good | “I don’t think you could have improved it really.” (P4) |
|  | use of video | “It was quite hard to get, yeah because in the video obviously the girl can talk a lot, so it's quite, yeah.” (P3) |
| Individual adaptations | adaptations for comprehension | “And we feel we're adapting- I don't know, I definitely adapted cause he didn't- he doesn't have that understanding for a lot of the game things.. like if you say, "What's this?" it just goes right over his head.” (P2) |
|  | adaptations to activities | “No but there was a card one [laughs]. There was one that wasn’t a matching game but we made it a matching game first..... And then we did what we should have done afterwards.” (P1) |
|  | adaptations to sequencing | “so we didn't- she didn't do the beginning middle and end, I showed her.. .. And we did that thing, where you know "This is the beginning", and then we wrote it together.” (P1) |
|  | many adaptations for my child | “You know, once we are sat there and we are doing it and he's well, and it's a great little scheme and I have to make a lot of alterations to it” (P4) |
| Theme 7: Capturing intervention effects | | |
| Capturing change | capturing child attempts | “Yeah, like attempts or something.” (P2) |
|  | capturing progress | “But it's hard isn't it? It's like how you record it?How do you- how do you measure it?” (P4) |
|  | measuring progress captures unexpected abilities | “And that was nice to see because she could show me what she knew, that I didn't know she knew.” (P1) |
|  | measuring progress in different ways | “Capturing progress and stuff..So when you get to the end and you're like, oh I know he's made progress but it doesn’t look like he has with these ones and zeros. And it's like, you know that they have.” (P4) |
|  | measuring progress with specific targets | “I'm going "No, no, no, remember, wings!" and he was going round but like, it just- it just made me laugh cause im thinking, I'm like I'm doing the monkey sign and then like oh yeah of course he's not relating that we are talking about wings here, cause we are onto the other two words” (P4) |
|  | parents’ perceptions of progress | “But yeah, maybe just another box, for us to put in, I think she's learnt this this week.” (P1) |
|  | using video captures progress | “he was tired that day and I thought "it aint gonna go well today." and he really engaged for some reason and he was getting right to the thingy and he saying things. and I was like "Oh! I'll do it". So I did it all, and then when I looked back its all gone in slow motion [laughs]. And I was like "No!" because he had, he really really did engage on that one, and again its nice to look back and think, well what signs he did or what attempts he made”. (P2) |

**S4b: Adaptations to PACT**

| **Category** | **Code** | **Meaning Unit Examples** |
| --- | --- | --- |
| Theme 1: Principles to support learning | | |
| Language and learning opportunities | language exposure is useful | “I don’t think he needs to answer them. You're just giving him opportunity to hear them.” (P1) |
|  | parent priorities for targets of intervention | “For-for me, for-for when I joined up to this, thinking it's for speech therapy, the activties, book, everything I love, but for me, I'm trying to get him to say the words so it's- when I'm trying to say farm I want.. to be saying how to ge- for me to get him to say fa-rm. You know like, how to say farm not just 'say farm', if that makes sense. ..how to, get him to do it rather than, he likes the books, he likes the games, but my aim is speech therapy, get him speaking, so how- do you know what I mean?” (P2) |
|  | repetition of language input | “Yeah, I think it's about exposing them to things. So the thing that makes- the first thing that sprang to my mind was that, when I was- when she was a baby and I was trying to teach her makaton and I was doing the signs over and over again and she just wouldn't do anything back. And then one day, she just signed 'more' and it was like a lightbulb moment and it's that thing that you're not expecting to see....the results straight away, you're sort of- you're exposing them to it and embedding the- it in to them so that it's gonna come out, whenever, in their own time. Like anything else that they do is in their own time.” (P1) |
|  | repetition supports development of understanding | “like I say she-she might not show me that she's understanding but it might be just seeping in in little drops that you know..but it might be just seeping in in little drops that you know.. That the story does have a beginning, a middle and an end.” (P1) |
| Relatable content | culturally appropriate materials are more recognisable | “the barn in the farm was like an american barn, like a red barn, and so, she's never seen an American barn before.” (P1) |
|  | impact of different lives on relatability | “Where as some kids have probably never seen some of these animals or.. It's just- each kid is going to be different aren't they? Like, you know, some kids have probably never been to a beach, if they live right in the middle of the country or something.” (P4) |
|  | relatable materials | “Yeah, I think just keep it as real as possible.” (P4) |
|  | relating to personal experience | “I'm just thinking, at the beginning it would be good to have like, um, a prompt to say 'you might find useful in this section'. Um, where it says 'Have you been to the beach before?', if they have, have a photo of them ready… .. Like pictures of them at the beach, pictures of them in the forest or whatever that, that kind of section at the beginning. Because, it's relating it to their life. Um, and obviously if you've, you know as an adult, if you've done it yourself you understand it a lot more than if you've not.” (P1) |
| Repetition | more opportunities to reinforce vocabulary over time | But that one, if you went back to say like, sticker and you might not want to do this, but something like stickers, extra things that belong on the beach, and then the ones that didn't belong on the beach belonged in the forest from the day before. So you're still capturing that- those words from yesterday.” (P1) |
|  | more repetition of activities | “Where, um, you’ve got each, um forest, you could do the same activities but with a different page of the book. Like the snow one, or the mud one.” (P1) |
|  | repetition in everyday contexts to support learning | “It's the repetition, and I don’t know maybe if you put some ideas in, how you could, um, how-how could you further this if you have time, if you have time [laughs]. You know, what could you do round the house, what could you do in school and things like that.” (P4) |
|  | repetition is influenced by child's interests | “I'll get it out and if he has done it for a couple of days he'll go and get his own. And like, I'm like 'We're doing this one" and then he's like "No no" and he's getting his.” (P2) |
| The importance of active involvement | active choice-making | “Or I can offer her a card and she feels like she's got a choice of which one…” (P1) |
|  | active involvement supports engagement | “So when we play bingo, I gave her a choice, we turned them over, it's that doing something that keeps her.. So ones where you're doing something? Yeah. But ones where it's just point to, or show me..” (P1) |
|  | active involvement supports learning | “Yeah, or, cause the next one is drawing other things you might find on a beach, well no. You'd need two lots of cards, like sorting, what would you find on a beach and you wouldn't. And that's what I put for that, um, tree game, is having a sorting of what's forests and what's not forests. Cause sorting is- was one that they like and…” (P1) |
|  | active involvement supports motivation | “Just less motivating aren't they? And less like…” (P4) |
|  | active involvement supports understanding | “Yeah, that beach one is a nice activity but she wouldn’t, um.. She wouldn't get it. Unless it was stickers.. Unless it was 'sandcastle', right put the sandcastle sticker on the sandcastle.” (P1) |
| Use of text | accessible text | “the other thing, was like having the font bigger for them, um, and having them on white- black writing with white background, so she can see it clearer. Um, cause she's got a little bit of a stigmatism, but she doesn't need glasses, but it's that.. that thing that like Maggie Woodhouse says, that they see through like a, almost like a..Like a sheet of plastic or something, they don’t see as clearly. So having the printed materials bigger and clearer…” (P1) |
|  | simplify text support | “I'd say the same about the definitions underneath, I thought that. I didn't think to bring it up but I did think it at the time. I thought, don't really need that there. It’s just confusing information isn't it? That you don't need…. Yeah, so could put it on the back like you said. Or have it somewhere else, or, or maybe we should know definitions.” (P4) |
| Visual resources | using objects supports understanding | “I tried lining up things, you know like objects? Saying "Which one is first?" (P3) |
| Visual supports | abstract pictures | “I thought, lots of pictures were just, I think you mentioned it, I think some of them were just too abstract.. with the word, like I think it was the dog jumping through a hoop..All he would do was sign dog. And the farm, all he would do would just sign sheep.” (P3) |
|  | careful selection of pictures to support learning of concepts | “Was it the drawings? Where all the drawings were different as well and you had big and small but they were…not the same pictures. As well, so he was kind of like, "What am I meant to be doing here? Am I meant to be talking ab-", you know it was like, they needed to be identical. And that would have been OK being illustrations but if they'd been three identical things or just two, I think you talked about.... that when you came round, um but he- I had to throw- right not doing that one, wouldn't have that one at all. I think it was like a blue, green and a yellow train, was it or something? And he was supposed to be saying which one was the biggest, and I think the colour change was a distraction for him.” (P4) |
|  | drawings are less accessible | “because in that one, the farm in the gingerbread book, they were drawn animals.So, because the drawn animal wasn’t the same..She sometimes didn't.. Where as if it had been a picture, she- I think she might have got it a little bit.” (P1) |
|  | eliciting target from a visual when the concept is abstract | “Because they kind of like, I can't remember what the prompt was, something like, "Where is the girl in the queue?" and he just went "There" And he pointed to the one highlighted. But it wasn’t'- because I'd tried to do it.” (P3) |
|  | impact of visual recognition | “Well do they know which end the queue starts at? Can they understand that that person is facing that way so therefore theres the.” (P4) |
|  | photographs are accessible | “I think make it as easy for them as possible.” (P4) |
|  | photographs are effective | “I think the photographs have been great… Yeah, I think Child4 last night surprised me, he was saying 'bird' the other day to the parrots and then as soon as he saw the photo with the parrot with the word Parrot.” (P4) |
|  | photographs are motivating | “But yeah photgraphs are great. I think all of those matching games, um. Really really motivated by photgraphs.. ..and high quality photographs. So, I think they were really good.” (P4) |
|  | signing support | “That's- actually that's I was going-I was going to say, if you could provide some of the signs [laughs]. Cause, I don't know is there a sign for full and empty?” (P3) |
|  | signing system | “It would be finding the one that most people use” (P1) |
|  | symbol support | “I was- I was thinking makaton symbols might be helpful, but I don't know how you'll do it.” (P4) |
|  | abstract concepts are hard to elicit from visuals | “And the kennel one, she would just sign dog, whether it was inside or outside. And with first and last, is tricky isn't it?” (P3) |
|  | careful selection of pictures for sorting activities | “Cause one was really similar, cause one, is it a field or is it not a field? Cause um, some of those pictures were confusing because, the ones that werent fields, say it was a river, you could see grass on it.And she just saw grass and her- when I did fields, she signed grass.” (P1) |
|  | objects and pictures support delivery | “go round the house and get stuff together. Or pictures of them at the beach, something like that.” (P3) |
|  | pictures plus words | “Yeah, or if- or if you had a separate thing, with the sandcastle, with a picture of the sandcastle and the word.” (P3) |
| Theme 2: Individualisation is key | | |
| Adaptation to the individual | flexibility for different abilities | “Yeah… could have some suggestions on how to take it down, yeah. It's just catering for that wide ability range isn't it? Cause it's so wide.” (P4) |
|  | heterogeneity in profile | “There’s just such a big variance isn't there, um, with their ability in Down syndrome children.” (P4) |
|  | core and optional activities | “Maybe some suggestions on what you could do, um to expand on this scheme, to reinforce. Some suggestions, just suggestions I think you need to stress, cause some- some parents are up to here aren't they, they can't, loads of kids and…” (P4) |
|  | incorporating familiar songs to support learning | “Yeah, cause even if you were gonna do it like an extra day, if you were gonna do the snow one, for us we would use Frozen. We'd use Le- Let it go the song to show, you know the cold and stuff. But it's things in popular culture isnt it? That you could just, use to support.” (P1) |
|  | individualised resources | “So you could do the- you could make your own, if you had the time.... Like the sorting ones or things like that.” (P1) |
|  | step up and step down activities | ‘But then an- and the other way as well, if your child's not got it, what're you gonna do to take it a step back? So like a step forward and a step back, so if they totally don’t get that, how can you simplify it?’ (P1) |
| Theme 3: Adaptations to PACT-DS structure | | |
| Book choices | book choices - familiarity supported engagement | “I think they're also um, the two we've just talked about there, the Handa and the other one, um, the Catapillar, they're kind of done in school. So Child4 had done those already and he was like, straight away I said oh you know, he was saying "Oranges!.. Tree!". So he knew that they fell from the tree. So he'd done that, and I think, yeah that helps doesn’t it?” (P4) |
|  | book choices - parental preferences | “I think they've been OK so far. I think they've been alright, I've preferred some of them than others.... But I think they're alright.” (P4) |
|  | book choices - quantity of text | “What was nice about the Caterpillar and Handa was, there’s a short amount of text on each page.” (P1) |
|  | book choices - relatability | “See, we live near Chester Zoo so we've got, so Zoo animals in Handa was great for Child4 cause he goes to the Zoo all the time.” (P4) |
|  | book choices - representation of Down syndrome | “No book, I know it's not for them, with a child with Down Syndrome in. So that might be a nice one to include.” (P1) |
|  | book choices - visual complexity | “I think the Usborne ones, the Gingerbread one and the Vet one were a bit busy.” (P1) |
|  | book choices- interactivity | “It's just quite an interactive book isn’t it? It's, it's got lots of nice words and stuff.” (P4) |
|  | book choices -shared experience with peers | “I think the choice of books is good because like you say it's- it's, the whole point is about developing their language skills, and if they, um, can interact with other children their own age at nursery or at school about a book then surely that's part of what you want to happen.” (P1) |
| Vocabulary | abstract concepts | “I think the change, is it the change one as well. And the- and it wasn't even related to the story at all, he just, he just was signing rain....cause he that’s what he can…” (P3) |
|  | opposites help learning concepts | “I think it's good to have them in the pairs as well, isn’t it? Cause- cause it's the opposites, the opposites, I think just work so much better. It's like when we're doing empty and full, it's- it just works well cause you can show.. It's "Oh yeah, OK I get it now" rather than just an.. empty box . That's what he would say "it's a box." (P4) |
|  | parental preferences on vocabulary | “I think scared at the end, but we'll come to it, but I think- I think scared's OK but I think that that's the only day where I've gone.... I don't like that activity. And you don't want to introduce fears [laughs] you know” (P4) |
|  | range of word classes | “I think they're good, I think what you need is like two.. Two nouns that are very much 'this' and 'this' that they can grasp, but then other, more, like verbs. Because for- for our children, like prepositions, they're not, well they probably will know them, when they begin to talk they don't use prepositions.” (P1) |
| Sequencing | challenges of sequencing | “Child4 doesn't really get the order, he-he was able to say a sentence-ish for each one, sometimes it was just.. Walking on head, that's what he said you know for-for how they're walking on head [laughs]. And I was like- so then I made it into something but he- he would have no idea how- like middle? No.” (P4) |
|  | consistent structure signalled end of sessions | “And even though she didn’t get the beginning, middle and end it was a nice way, because she knew that that was the end of the session.” (P1) |
|  | embed sequencing concepts in shared reading | “Could just say, this is the beginning of the book.. Rather than doing it when we've read it.... If that makes sense?” (P2) |
|  | phased integration of sequencing concepts | “Where the turning point of the middle of the story is… Well maybe they will get it by the end of 30 weeks?... Yeah.. Could start with first and last, you know like you said, an then introduce middle much later.” (P4) |
| Existing activities that work | appropriate choice of vocabulary | “I think they're genuinely quite good.” (P3) |
|  | extrinsic rewards | “And she could get a sticker” (P2) |
|  | importance of shared reading element | “You're not just reading it are you? You're asking, you're doing a lot of the work in that orange bit, that's almost like the main part. And I'd say these are like, the- all of this is almost like plenaries isn't it? Consolidating.” (P4) |
|  | over and under are effective activities | “Some of the stuff in here is just great! Like some of it, you know like over and under. I mean, we're working on that anyway. Some of it is like "Oh god, no".It's-it's like such a mixture, like some of it- some of this, even though it's not been written for children with Down Syndrome, it's absolutely brilliant. ... the over and under game, it's great photographs and, you know, that's the kind of thing that they do in speech and language therapy, so.He'll- he'll be able to do that, or he'll, he might not get them all but it's really good.” (P4) |
|  | positive opinion about inclusion of shared reading | “I like the reading, I like the books.” (P3) |
|  | using songs supports engagement | “Child3 liked the farm one cause he was singing old Macdonald..He liked singing it.” (P3) |
|  | topics that support engagement | “Definitely more food in. Food and animals you're onto a winner.” (P4) |
| PACT resources | lots of resources | “Yeah, there’s a lot of little cards” (P2) |
|  | organisation of activities | “I keep losing all the cards, whe- when you say put them on the word collector, like how do I fix them to it? I know that's stupid and I should have a method at home but I don’t. So they just keep getting- falling off, I tried to tape them on.” (P4) |
|  | reduce parental load with resources | “Because, I'm quite functioning but like, sometimes I'm like..You know, I've got to cut all the blimming holes out...You don't, you don't want that do you? You don’t want that, you want..X, what you havent got to do everyday, it's all done for you, it's all perferated, it's not going to rip when you pull it out.” (P4) |
|  | suggested objects to support topics | “Or saying at the beginning, a list of things that would help you. So if- if at the beginning it said "This week you could use a bucket and spade if you've got one" like that, not go out and buy it.... But if you've got a bucket and spade. If you've, for the forest, if you’ve..” (P1) |
|  | limitations of existing resources | “The one- the one in the vet one with the, with the card of.. I can't remember, you had to fill it in.I'd get rid of that.We did it in our own way, we got a stethoscope, we got our dog and we did bits but it was pointless..Cause the writing down was for me, or for- she wasn't learning anything from that one.” (P1) |
|  | careful selection of pictures for concepts | “It's your interpretation of scared as well because there are some pictures which are like scared of spider, where as Child1 absolutely loves playing with spiders.” (P1) |
|  | adaptations needed for bookmarks | “I do use them, or I do adapt them. and I think sometimes it's useful, sometimes it's not, sometimes you had to adapt it a lot sometimes you just have to tweak it or you have to repeat a bit. But it's good, it's good.” (P4) |
|  | bookmarks helped delivery | “it did sometimes prompt me to remember to say "Oh yeah", or prompt me to exaggerate when I got to the special.. The special words and stuff.” (P3) |
|  | bookmarks helped preparation | “I did read them before, I didn’t really look at it while I was doing it” (P3) |
|  | bookmarks were not used | “I didn't really look at them.” (P2) |
|  | losing bookmarks | “Yeah, I'm just thinking, is it another thing to faff with? You know, I lost yesterdays, don't know where it went, I was literally just sat next to Child4 and it had gone. Vanished! I was like "oh, where’s the bookmark?" (P4) |
| Theme 4: Recognising the unique role of parents as implementors | | |
| Flexibility | flexibility - following child's lead | “Yeah and if she's into the game we'll spend more time.... On the game.” (P1) |
|  | flexibility - timings of activities | “I think as well, because they've all got times, that puts you.. Cause you want to get it done, and the fact that.... Trying to do, or even just say, yeah...It depends what's going on at the time doesn't it?” (P2) |
|  | more flexibility in duration | “Could try it with more days…” (P3) |
|  | more flexibility in schedule | “You'll say 'Ideally, over a week, if it takes up to 10 days' you know, to allow.. Yeah.” (P4) |
|  | more flexibility in schedule for repetition | “Cause then you could repeat a step..If it's totally new to them.” (P3) |
|  | prioritisation of activities | “Sometimes I didn't read the book, I've mentioned that before.. Especially if it was one she'd read before because then that meant we got through more, but it depends on whether your child would need to read the book” (P1) |
| Parent delivery | simplify delivery of PACT | “Oh, I don’t know, I just- I'm just … I'm thinking that these parents are going to be, some of them, are gonna be wanting it as simple as possible, to make the maximum impact.” (P4) |
|  | using objects that belong to parent are motivating | “She thinks she's using my stuff, like "Oh I'm getting into your stuff there" do you know what I mean.... and that gets her more interested, um.” (P1) |
| Generalising from PACT to other learning opportunities | extension to other learning activities | “But I think that's what the, I was- I think I was saying to you that um when they've done a book, a- another book, it's nice because then you can think of activities that you could do with that book.” (P1) |
|  | potential for extension to other books | “I th- this whole programme opens doors for other books that you could use with them, that are more specific to your child.” (P1) |
| Parent training | parent training - signing | “Yeah, and I think you can't see that all parents are gonna have sign, I mean others might do here but.” (P4) |
|  | parent training about individual adaptations | “But also it would have to be really stressed in there, like, you don’t have to do this bit [laughs]. You know, don't feel like you have to do this, it's just in case.” (P4) |
| Parent perceptions of child abilities | comprehension of questions impacts accessibility | “I just worry, cause with us, like I say he can't answer any questions apart from the yes or no.” (P2) |
|  | impact of language needs on engagement | “But even them questions, he's not gonna answer any of them to me.” (P2) |
|  | level of conceptual understanding influences delivery | “They don't have them concepts, do they? They don't know, so, it's a-it's a, yeah some parts are really good aren't they? And others parts are a bit like "ugh!"… they just haven't got them at all.” (P4) |
|  | level of understanding impacts accessibility of activities | “Looking at these, like the games, I don’t think Child2 would understand any of these” (P2) |

**S5 – Data-driven Adaptations to the PACT Programme**

| **Category** | **Parent Feedback (focus group)** | **Unstructured Observation and Discussions** | **Adaptation** |
| --- | --- | --- | --- |
| **Repetition and consolidation** | *“The extra activities in Week 5 should have more games to focus on the abstract words to help consolidate.”* | Parent revisits previous words in themes in activities (e.g. ‘wing’). | We added (optional) extra activities in Week 5 and more opportunities to consolidate learning. |
|  | *A lot more repetition for children to learn the words and concepts (e.g., “first/last”). Ideally do things (including certain activities) again and again to consolidate.*  *“He [child] likes the repetition.”* | Parent noted throughout the need to keep revisiting learning for consolidation – takes longer to learn something but also needs repetition to check child hasn’t “lost it.” | Special words are now targeted twice, across two books, and we have repeated certain activities to support consolidation. |
|  | *“He really just wanted to match the pictures and he felt like he was doing well at that, cause he was linking it to the book.”*  *“Simpler games, matching would be easier, repetition for games.”* | Matching game – Child responding with sign, “rabbit”, “cat.”  Parent building lots of excitement and anticipation for the card (matching) game – child enjoys this!  Child matches cards independently, picking them up, placing them down. Parent supports gently when needed e.g., by guiding her arm or saying “look again.” | We limited the types of games used in the activities, instead repeating easier games (such as matching activities) across the programme. |
| **Reducing time pressure** | *“I think you need to call it something else, I think you need to sort of say 'Ideally this would be done over a week.’”*  *“Some days were busy or if he had to attend nursery were difficult to fit it in. Needed to try and do it when he was fresh and not at the end of the day.”* | Parent noted that pace [of the programme] is ok but would be useful to have more revision before moving on. | We increased the amount of time spent learning a new word – 4 words are now leaned across two books (5 days per book) and introduced a further recap day on Day 5 to allow flexibility.  We also removed the introduction and reward as named components, and removed stated timings for each component. |
| **Tailoring to individual needs** | *“Being able to change it, and also having the expectation that we might not finish... the section, the way I thought it was going to go, and that's OK.”*  *“Could give us the option to add something to make it harder or reduce the activity to make it a little easier.”* | Parent is introducing upwards and downwards extension activities herself | We added suggestions for upwards and downwards extensions for activities.  We built more flexibility into the programme – a recap day on Day 5, consolidation week with a flexible day that can be used to reinforce words already covered or catch-up on missed ones according to individual needs. |
| **Smaller steps for learning** | *Perhaps 5 words in 5 days can be too much to focus. So reduce and if a child is struggling on 2/3 we can repeat.* | Parent notes that simple activities which are repeated would be better than lots of different activities – when the child understands the activity and what they need to do they are better able to learn the content (e.g. target word) e.g. parent notes they often sing ‘if you’re happy and you know it’ song and as child knows it they can bring in extra instructions that he will then try and do. Because he knows the routine of the activity he feels able to join in. | We reduced the number of words to 4 words over 10 days, which are repeated over 2 books. |
| **Supporting engagement with the programme** | *On that note of behaviour, I was just wondering whether we can have like a tick box thing for them? like a working for board, on the side.*  *I think it's nice to just talk about the picture, and then you could give them "Are there any words I need to put in it?". Cause, she might sign bear, she might sign dog and then I could build it around that…* | Child was engaged – vocalising, looking at the pictures, pointing to and touching the pages.  Parent reads the book and uses lots of really good prompts to support child engagement and interaction – including open ended questions, sentence starters, sounds (What is this? mmmm – moon), pointing to pictures. | The Stories activity has been overhauled to include more visual resources as choice options providing additional scaffolding and supporting engagement.  We included an optional visual timetable. |
| **Visual support** | *Yeah, I think just keep it as real as possible…. having the font bigger for them and having them on white- black writing with white background, so she can see it clearer”.*  *“I think including the sign…when he's learning I think it's important”.* | Parent shows the child the special word on the word card and they find it in the book.  Parent also…uses signs and interaction to support his understanding e.g. rubbing his tummy when saying ‘hungry.’ | Photos are used rather than cartoons/drawings where possible. Text is made bigger and black on a white background.  We have included the Makaton sign for each special word. |

**S6 – Figure S6.1: Time spent on intervention delivery per session in Cycle 2.**


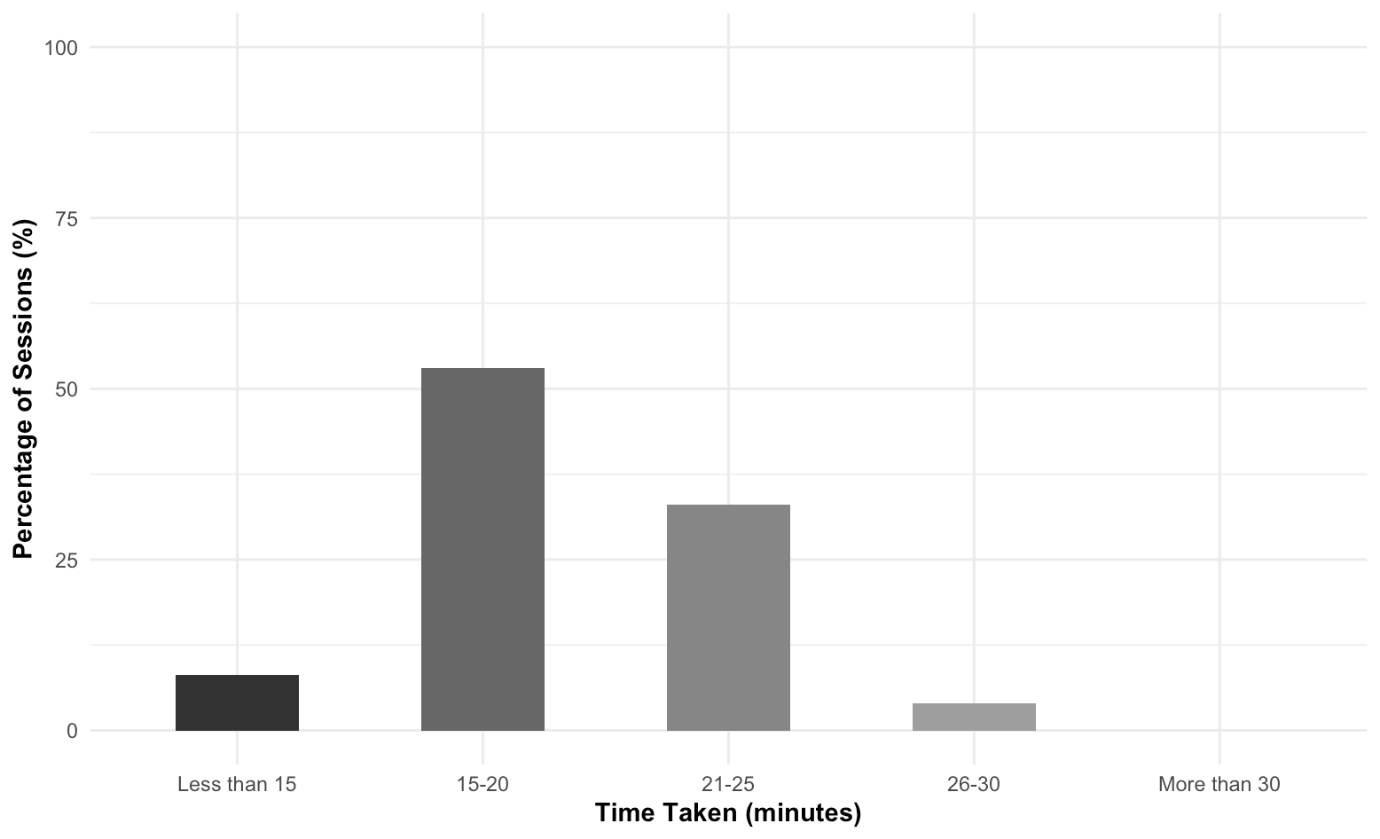


Figure S6.1: Time spent on intervention delivery per session in Cycle 2.

**S7 – Focus Group Analysis – Cycle 2**

| **Theme 1: Parental views on revised PACT-DS** | | |
| --- | --- | --- |
| **Category/subtheme** | **Code** | **Meaning Unit Examples** |
| PACT-DS is overall better than PACT | Repetition of activities good for learning | “because a lot of the, um, ways of doing like the activities were the same so I wasn’t teaching her how to do a new activity, it was just the learning of the words and the content” (P3)  “it's like sticking with that same sort of routine and not introducing all these new games, even- although they were fun in the PACT, I don't know whether he was learning that much during it” (P1) |
|  | Accessibility/Better paced | “this felt like it was accessible” (P1)  “Whereas PACT, typical PACT, was like fast paced and it was like, oh- oh we're doing something else now. You know, it's just like, he wasn't learning anything really, I don’t think [laughs] in that one, just wasn’t suited was it? But yeah, no it was like vastly better [laughs] like didn't want to cry at the end of doing these” (P1) |
|  | Adaptability | “easier to adapt individually” (P3)  “here was wa- suggested way to help you adapt, which was good” (P1) |
|  | Experience of success | “Less sort of feeling a failure, you know for us, as well as them” (P3; P1).  “I thought I don't know if he's going to get this and I wasn't even entirely sure if I got- totally got what I was supposed to do [laughs] but then he really surprised me” (P1)  “Middle, no no, he doesn't know what that means, I- yeah, but beginning and end, I think he's- think he's grasped that from this scheme”.  “he already knew house but he was saying it clearer”  “you know, one I kind of noticed first is family..using that loads now..  “Child2 as well... that word really worked”  “I realised that Child2 could read 'Mum' without a picture which I didn’t know”  I said "Which one’s sad?" and she said that "Cry" so she got- there were some nice surprising bits where I thought “she's never going to get that” and she did. It’s funny how they get the emotions even though they're quite high level”  “So, even though, do you know- do you know what I mean, I think- I think do that and then.. He really got beginning in the end, because I said "where in the story..", actually as- I wrote it on the- on the sheet- but I said "Where in the story does this happen?" think it was in Monkey Puzzle and he said ''gegginning'' |
|  | Makaton symbols help | “Makaton are discreet symbols and they're simpler and they're black and white. I'm quite a big advocate for Makaton so I feel it's important.” (P1)  “incorporate Makaton into this, it’s like, I think it’s really crucial…”  “It was handy having the picture cause I didn’t know what, I didn’t actually know what count was”  “I know they can be a bit funny, can't they, Makaton?”  “you have to permission. Yes, cause you- it's quite a lot of money really” |
| Challenges of PACT-DS (discuss how these are not specific to PACT-DS but will be applicable to any language intervention) | Difficulty representing challenging concepts visually | “Where we struggled was where you had, like a picture of a lady, just a random lady, and have mum under it, so I'm thinking that's not his mum, that's not any mum he's ever known, that's just a lady, you know” (P1)  “I suppose the way to get around that is to put the pictures from the story of the Auntie and.. Pictures from the story would work”. (P3) |
|  | Tricky concepts | “When food is finished and that, but the- I think she's learnt beginning from this, but she's still not grasping middle, which is, to be expected I think”.  “Child3 got confused with count and counting but count and numbers..Cause she's not very strong with maths at the moment, and I don't think she understood count.. |
|  | Effort and preparation time required | “There was a lot of pre-preparation involved in this wasn't there? It was kind of like, I couldn’t just open it and do it, I had to like, the night before, I had to- that's a lot, you know that's a lot of effort for me” (P1) |
| **Theme 2: Further adaptations** | | |
| **Subthemes** | **Code** | **Meaning Unit Examples** |
| Content | Teaching concepts (Fewer words, fewer concepts to learn and more repetition ) | I think having those going over longer, less concepts to learn, less words to learn, so you're repeating them. (P1) |
|  |  | “starting with you know like on the maths for life programme where she starts with 'big' and small'.. ..'More' and 'less', I think maybe having those as first concepts...To make sure, 'hot' and 'cold', 'fast' 'slow', you know all those simple comparisons” |
|  |  | “while she did struggle I think adult and child is a really good idea to teach in that you te- it- it leads on to sort of not stranger danger… Maybe it should just come a bit later in the programme” |
|  |  | “you know what I mean with the simple comparisons don't you? Cause that helps with numeracy” |
|  |  | “Over and under, I mean that’s brilliant…So, yeah, more prepositions” (P1) |
|  |  | “kids with Down Syndrome do- can find rhyming hard, my- Child1 loves rhyming [laughs] absolutely loves it” |
|  |  | “some sort of like pegs on a washing line. Because I could put it, you know that way of s- of sort of teaching where you make a mistake on purpose” |
|  | Relating the words learnt to the real world | “So you’ve got more chance to, um, show them in the real world. (P1)  To, you know, to go, oh hang on, we're working this this week. Yeah, and it also shows that its not just related to that story…” (P3) |
|  | Hands on activities | “some sort of like pegs on a washing line. Because I could put it, you know that way of s- of sort of teaching where you make a mistake on purpose” (P3) |
|  | Presentation of materials | “obviously because you've adapted them yourself, when this project is finished, will the adapted scheme be like the original scheme, or not? In that it's printed in those books and things, because I think what made it easier in the original one is sort of, the colour coded sections, cause when you've just got a photocopied sheet in black and white to read, and you're trying to skim read and you're feeling pressure..” |
| Flexibility/adaptability | Step up and step down tasks | I didn't ever once look at the extra challenge bit and go "Oh he's not going to be able to do that" and feel bad about it.. .. Cause-cause you also had one to make it easier (P1) |
|  | Programme to accommodate heterogeneity of DS population | “our kids have got- they're so different .... That the range of them...Even the same age” (P1) |
| Adaptability/Choice | Programme need to be easy to adapt to child's context | “I think by keeping it simple, as well, it gives you more leeway to adapt it to how you want. So, if you had something that you could bring in, like um pictures of your own family or something, you could do that” |
| Timing of delivery | Flexibility in delivery | “Allowing for a holiday week or two..Yeah, maybe just, yeah. (P3) Cause some of these aren't, we aren't doing them over a week, are we?” (P1) |
|  |  | “a lot of these children are gonna be younger and won’t be in school so it won’t affect them, but I don’t know if you can try and plan it so it's the very start of term” |
| **Theme 3: Barriers and facilitators of successful implementation of PACT-DS** | | |
| Factors affecting programme success | Repetition is helpful | “It's just repetition I suppose, isn't it?”  “The repetition was great though, like XXX with the first, the other 2 books and by the end like he- he wasn't saying, trying to say over and under at the beginning or sign it and by the end he was doing over and under” |
|  | Book choice/preference | “I abandoned that one story because that was Anna Hibiscus, he hated it, absolutely hated it. He wouldn't read it after day 3, "No, no, Bear Hunt, no!" [laughs]”  “New House was really apt for us because we'd just moved house, so it was like a relatable life event”  “You know it just felt like, ahh, and it's such abstract concepts for Child1, to think of Aunties living in the house as well, like that doesn't happen in the UK does it? Well it probably does in some families, it doesn't in our family, doesn't in the majority of families, and yeah. I didn't feel like it added anything to the adult and child concept”  “there's another world out there isn't there? So they need to be exposed to it, like everyone else does.. . And so, but it's finding that balance, you're right” |
|  | Things need to be simple | “I just feel like with our kids, everything needs to be quite simple for them to embed these” |
|  | Familiarity helps | “With our children, you need them to- to know what they're doing so they need familiarity” |
|  | Visual support | “Child1 is really good at generalising animals like he knows that that's an elephant, that's an elephant, that's an elephant and that's a monkey, that's a monkey. But when we came to create the sentences because that monkey wasn't the monkey out the book he was disinterested. He just wanted to create the sentence from the story, so we abandoned the pictures in creating the sentences, and I just said "What's happening here?" and helped him along. So yeah, in that case I kind of felt like ditch the images”  “But then you go back to what we said initially, I think I said "Use photographs they're much clearer!" but it's so difficult”  “I know, I know, but it's a mix isn't it, because the photographs do work well when you're trying to categorise, so with the adult and the child and you're putting them in piles, or you know another concept where you had in the original PACT, where it was field or farm, photographs work great there. It’s just concepts I think where they don't” |
|  | Resources | “I think that that sheet there would be better as an A3, because the pictures didn't always fit in and she wanted to stick them on herself and then my OCD was like "It's not straight" "It's not fitting" and we've not got enough space for the next one, and I think for it to be more child friendly and interactive, just have it A3 so that we can fold it in half and stick it in our pack because, unless I'd thought ahead and trimmed...”  “You know, if you were able to get it all nicely printed, I mean a lot of this would be, problems might not exist”  “Loved the stickers” |
|  | Change of focus helps keep interest | I do like a good washing line. Yeah and seeing it up there. It also just changes that focus from always looking down to looking up and just, I think it sort of like refreshes their interest, |
|  | Parental awareness of a different learning trajectory | “I think our kids don’t necessarily learn things in the same order so they're quite in tune with, you know….It's like they don’t necessarily do things in the same order do they?” |
|  | Parent delivery | “you’re going to end up with, um, data from families like us, you know where the parents are very invested in their children and that’s the only thing. I think you'll probably get a lot of drop outs from possibly families that haven't got the capacity to do it”  “we only have after school”  “The holidays were hard though, because I mean you might as well write those out…” |
